# Vitamin D and brain health: an observational and Mendelian randomization study

**DOI:** 10.1093/ajcn/nqac107

**Published:** 2022-04-22

**Authors:** Shreeya S Navale, Anwar Mulugeta, Ang Zhou, David J Llewellyn, Elina Hyppönen

**Affiliations:** Australian Centre for Precision Health, Unit of Clinical and Health Sciences, University of South Australia, Adelaide, Australia; Australian Centre for Precision Health, Unit of Clinical and Health Sciences, University of South Australia, Adelaide, Australia; South Australian Health and Medical Research Institute, Adelaide, Australia; Department of Pharmacology and Clinical Pharmacy, College of Health Science, Addis Ababa University, Addis, Ababa, Ethiopia; Australian Centre for Precision Health, Unit of Clinical and Health Sciences, University of South Australia, Adelaide, Australia; South Australian Health and Medical Research Institute, Adelaide, Australia; College of Medicine and Health, University of Exeter, Devon, United Kingdom; Alan Turing Institute, London, United Kingdom; Australian Centre for Precision Health, Unit of Clinical and Health Sciences, University of South Australia, Adelaide, Australia; South Australian Health and Medical Research Institute, Adelaide, Australia

**Keywords:** 25-hydroxyvitamin D, vitamin D, Mendelian randomization, dementia, stroke, magnetic resonance imaging, UK Biobank, prospective cohort study, brain volume

## Abstract

**Background:**

Higher vitamin D status has been suggested to have beneficial effects on the brain.

**Objectives:**

To investigate the association between 25-hydroxyvitamin D [25(OH)D], neuroimaging features, and the risk of dementia and stroke.

**Methods:**

We used prospective data from the UK Biobank (37–73 y at baseline) to examine the association between 25(OH)D concentrations with neuroimaging outcomes (*N* = 33,523) and the risk of dementia and stroke (*N* = 427,690; 3414 and 5339 incident cases, respectively). Observational analyses were adjusted for age, sex, ethnicity, month, center, and socioeconomic, lifestyle, sun behavior, and illness-related factors. Nonlinear Mendelian randomization (MR) analyses were used to test for underlying causality for neuroimaging outcomes (*N* = 23,901) and dementia and stroke (*N* = 294,514; 2399 and 3760 cases, respectively).

**Results:**

Associations between 25(OH)D and total, gray matter, white matter, and hippocampal volumes were nonlinear, with lower volumes both for low and high concentrations (adjusted *P*-nonlinear ≤ 0.04). 25(OH)D had an inverse association with white matter hyperintensity volume [per 10 nmol/L 25(OH)D; adjusted β: –6.1; 95% CI: –11.5, –7.0]. Vitamin D deficiency was associated with an increased risk of dementia and stroke, with the strongest associations for those with 25(OH)D <25 nmol/L (compared with 50–75.9 nmol/L; adjusted HR: 1.79; 95% CI: 1.57, 2.04 and HR: 1.40; 95% CI: 1.26, 1.56, respectively). Nonlinear MR analyses confirmed the threshold effect of 25(OH)D on dementia, with the risk predicted to be 54% (95% CI: 1.21, 1.96) higher for participants at 25 nmol/L compared with 50 nmol/L. 25(OH)D was not associated with neuroimaging outcomes or the risk of stroke in MR analyses. Potential impact fraction suggests 17% (95% CI: 7.22, 30.58) of dementia could be prevented by increasing 25(OH)D to 50 nmol/L.

**Conclusions:**

Low vitamin D status was associated with neuroimaging outcomes and the risks of dementia and stroke even after extensive covariate adjustment. MR analyses support a causal effect of vitamin D deficiency on dementia but not on stroke risk.

## Introduction

Low 25-hydroxyvitamin D [25(OH)D, an indicator of vitamin D status] concentrations are common, and the prevalence of severe vitamin D deficiency (<25 nmol/L) ranges from 5% to 50%, depending on location and population characteristics (1). Vitamin D is a hormone precursor that is increasingly recognized for widespread effects, including on brain health (2, 3). There are various mechanisms by which active vitamin D may affect the brain, including the regulation of neurotrophic growth factors, influences on inflammation, and thrombosis (4, 5). With a growing interest in identifying modifiable risk factors for dementia and stroke, vitamin D has become an attractive candidate, as supplementation, diet, and sunlight exposure can maintain adequate serum concentrations (6).

Several aspects of brain morphometry can reflect cognitive decline and neurocognitive disease (see **Supplemental Table 1** for a brief review). The association between 25(OH)D and brain morphometry has been mostly investigated in cross-sectional studies but with promising findings (7). A systematic review conducted in 2014 concluded that vitamin D depletion is associated with lower total brain volumes, whereas associations with subvolumes were more mixed (7). Several studies have been conducted since this review, some of which suggest associations that are the strongest for individuals with vitamin D deficiency, such as those who have been institutionalized or hospitalized (8–11). Interestingly, higher 25(OH)D concentrations have been associated with brain markers reflecting cerebrovascular disease, including larger hippocampal volumes (11, 12) and a lower prevalence of white matter hyperintensities (8–10). However, most of the studies on brain morphometry have been relatively small, and causality is yet to be established as 25(OH)D concentrations have been measured around the time of brain morphology assessment, and these associations could reflect confounding or disease-associated differences (“reverse causation”). Many prospective studies are looking into the associations of 25(OH)D concentrations with dementia and stroke, some of which suggest a threshold effect, in which the associations are the strongest or restricted to those with the lowest concentrations (13, 14). However, the causality of the association between vitamin D and dementia has not been confirmed, and the few randomized controlled trials (RCTs) have not provided convincing evidence for the role of vitamin D on dementia or other related outcomes (2).

**FIGURE 1:**
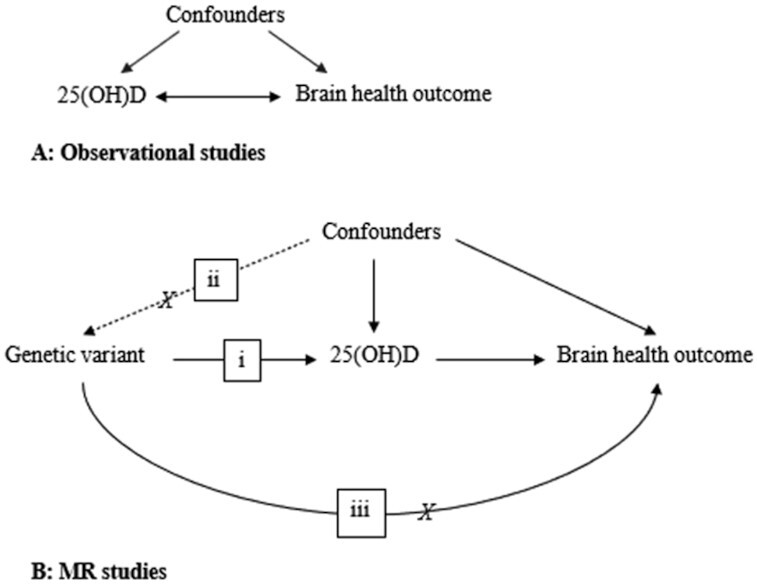
Characteristics of observational and Mendelian randomization (MR) studies. Observational studies (A) may be affected by confounders and reverse causality. MR studies (B) analyze the association between genetic variants, which are proxy indicators for the exposure [here, 25-hydroxyvitamin D (25(OH)D)] and the outcome to provide proof of principle for underlying causality (15). The diagram illustrates the core assumptions in an MR study, where genetic variants (i) should be associated only with the exposure, (ii) should not be affected by the confounders, and (iii) should not associate with the outcome through pathways or phenotypes other than the exposure.

Mendelian randomization (MR) is a genetic approach that allows testing for underlying causality when RCTs are deemed infeasible or unethical (15). In an MR study, genetic variants, typically single-nucleotide polymorphisms (SNPs), are used as proxy indicators for the exposure (15). Given that SNPs are randomly assigned and fixed at conception, they do not change in response to our behavior or disease experiences, thereby reducing methodologic problems related to confounding and reverse causality (**Figure 1**) (15). This approach has been used in at least 3 earlier studies investigating the association between 25(OH)D and the risk of Alzheimer disease, which provided some support for an association (16–18). However, causal evidence for an association between 25(OH)D and stroke risk is inconclusive (19, 20). In addition, all MR studies in this area, so far, have assumed linear associations. This type of approach is likely to miss an association in the context of a strong threshold effect, where the association is restricted to the correction of nutritional deficiency.

In this large-scale prospective study, we use information from up to 33,523 participants from the UK Biobank to examine the association between 25(OH)D concentrations with a range of brain neuroimaging features. Extending the analyses to 427,690 participants, we also examine the associations with the risks of dementia and stroke, for the first time, to our knowledge, using the MR approach to test for the causal effects of increasing 25(OH)D concentrations in the context of severe vitamin D deficiency.

## Methods

### Study population

UK Biobank is an ongoing prospective cohort study of 502,504 participants aged 37–73 y (99.5% aged 40–69 y) at recruitment (21). Participants were recruited across 22 centers in England, Scotland, and Wales from 13 March 2006 to 1 October 2010 (21). During baseline data collection, detailed information about the participants’ socioeconomic status, lifestyle, and health was obtained from self-reported touchscreen questionnaires, computer-assisted interviews, physical measurements, and collection of blood samples (21). In 2014, the UK Biobank incorporated an imaging substudy aiming to conduct MRI of the brain, heart, and body on ∼100,000 participants (22). Data collection is still ongoing, and at the time of the data analyses, information for >39,000 participants had been released (23).

In this study, we excluded participants who had ≥2 members per family, missing or low-quality 25(OH)D data, or a history of dementia or stroke (**Supplemental Figure 1**). For the imaging subsample, we made further exclusions of participants with missing brain volume data and outliers, which were defined as any brain volume data ±3 SD from the mean brain volume. Our final analysis samples included 427,690 participants for disease outcomes (3414 incident dementia and 5339 incident stroke cases), with the brain neuroimaging subsample including up to 33,523 participants (Supplemental Figure 1). For our MR analyses, we further excluded participants without genetic data, those who had a mismatch between genetic and self-reported sex, nonwhite British participants, and participants with ≥1 member from each family. The final sample size for the MR study was 294,514 for disease outcomes (including 2399 incident dementia and 3760 incident stroke cases) and up to 23,901 participants for imaging outcomes (Supplemental Figure 1, **Supplemental Text S1**).

### Ethics

All participants provided informed consent before data collection by the UK Biobank. All data collection protocols for the UK Biobank have been approved by external ethics committees, including Northwest Multi-center Research Ethics Committee, the National Information Governance Board for Health & Social Care in England, and Community Health Index Advisory Group in Scotland (24). Ethics approval for our analysis was obtained from the UK Biobank Ethics and Governance Council and Human Research Ethics Committee of the University of South Australia, and all data have been deidentified for analysis.

### Vitamin D

Serum 25(OH)D concentration was determined from samples collected at baseline using direct competitive chemiluminescent immunoassay (DiaSorin Liaison XL), with the assay having a measuring range of 10–375 nmol/L and CV of 5.04–6.14% (**Supplemental Text S2**). 25(OH)D values below (*n* = 2654) or above (*n* = 2) the reportable limit were replaced with missing (25). Furthermore, given sample dilution issues, we excluded participants (*n* = 10,002) with 25(OH)D data from aliquot 3 (Supplemental Figure 1). For categorical analysis, 25(OH)D was grouped as <24.9 nmol/L, 25–49.9 nmol/L, 50–74.9 nmol/L, 75–99.9 nmol/L, 100–124.9 nmol/L, and 125–240 nmol/L based on literature and Institute of Medicine and Endocrine Society Clinical Practice guidelines (6).

### MRI brain volumes

The UK Biobank conducted MRI across sites at Reading, Newcastle, and Cheadle Manchester (22). Scanning was performed using a Siemens Skyra 3T scanner running on VD13A SP4 software with a Siemens 32-channel radiofrequency receive head coil (22) (**Supplemental Text S3**). Scanning was conducted from the top of the head to the neck using a 256-cm superior–inferior field of view (22). The protocol consisted of sagittal T1-weighted images [1 × 1 × 1 mm resolution, time of repetition (TR) = 2000 ms, time of echo (TE) = 2 ms] and T2 fluid-attenuated inversion recovery (FLAIR) images with fat saturation (1.05 × 1 × 1 mm resolution, TR = 5000 ms, TE = 395 ms) (26). All acquired images were preprocessed and then checked for quality (Supplemental Text S3) (22). Although total brain, gray matter, white matter, and hippocampal volumes were extracted from processed T1 images only, white matter hyperintensities were identified from both processed T2 FLAIR images and T1-weighted images (22). All brain volumes were calculated using FreeSurfer software and normalized for the head size using a T1-based head sizing scaling factor (scaled brain volume = brain volume * head size scaling factor) (22).

### Dementia and stroke incidence

Data on incident dementia or stroke cases were obtained by the UK Biobank from linkages to available national data sets, including primary care data, hospital admissions electronic health records, self-reported health information from touchscreen questionnaires, and the national death registry (27). Incidence of stroke or dementia was defined as any stroke or all-cause dementia cases after the baseline assessment and before the end of follow-up on 1 February 2020.

### Covariates

Information on all covariates was obtained during the baseline assessment, with full information provided in **Supplemental Table 2**. Covariates were decided based on theory and literature. Basic characteristics included the month and participants’ age at baseline data collection, sex, self-reported ethnic group, and assessment center location. Sociodemographic factors included Townsend deprivation index (23, 28), education and employment status, and lifestyle covariates, including the type of physical activity, diet quality (29), use of any dietary supplements (yes/no), and BMI (in kg/m^2^), calculated based on measured weights and heights and categorized according to criteria by the WHO as underweight (<18.5), normal weight (18.5–24.9), overweight (25–29.9), and obese (≥30) (23, 30). To minimize the possible influences by reverse causality, we further adjusted for sun exposure behaviors, including time spent outdoors in summer and winter, frequency of sun protection, and disease-related indicators, including long-standing illness/disability/infirmity and depression.

### Genetic instruments

The most recent genome-wide association study (GWAS) identified 143 genetic variants associated with 25(OH)D using information from 417,580 European ancestry individuals from the UK Biobank (31). Of these, 122 were autosomal SNPs, of which we selected 35 common SNPs (minor allele frequency >5%) that had at least a nominally significant and directionally consistent association with 25(OH)D in an earlier independent GWAS by the Study of Underlying Genetic Determinants of Vitamin D and Highly Related Traits (SUNLIGHT) Consortium, which did not include the UK Biobank (32) (**Supplemental Table 3**). We extracted these variants from the third release of imputed genetic data by the UK Biobank (33). For genotyping, 2 arrays [UK Biobank Lung Exome Evaluation for ∼50,000 participants and UK Biobank axiom array for ∼450,000 participants] with 95% marker similarities were used, and here, we adjusted for the genotyping array. Haplotype reference consortium, UK10K, and 1000 Genomes reference panel were used for imputation. Genotyping, imputation, and related quality control were carried out by the UK Biobank central team, with full details reported elsewhere (33). We calculated a weighted genetic risk score (GRS) using the 35 variants with weights taken from SUNLIGHT consortium (32) (**Supplemental Text S4**).

### Statistical analysis

Linear regression was used to investigate the association between 25(OH)D and brain neuroimaging outcomes. White matter hyperintensity was log-transformed due to skewness, whereas other brain volume indicators were normally distributed. Cox proportional hazards model was used to investigate the association between 25(OH)D and risk of dementia and stroke. The proportional hazards assumption was examined using Schoenfeld residuals and was satisfied in all models for dementia and stroke.

Adjustments were performed progressively, adjusting for additional groups of confounders with each new model. The basic model was adjusted for age, sex, assessment center, ethnicity, and month. Further adjustments were done for socioeconomic factors (education, Townsend deprivation index, and employment status), lifestyle (BMI, type of physical activity, diet quality, and any use of dietary supplements), sun behaviors (time spent outdoors in summer, time spent outdoors in winter, and sun protection), and illnesses (long-standing illnesses/disability/infirmity and depression). Models were weighted by (1 – kinship coefficient) to account for relatedness (34). For analyses involving brain volumes, we performed sensitivity analyses, also adjusting for the duration between the baseline and imaging study visits, and after excluding incident cases of stroke or dementia occurring before the imaging visit. We tested for 2-way and 3-way interactions of age, sex, ethnicity, and BMI in each of the associations and conducted further stratified analyses in the presence of effect modification. In these models, interactions were based on likelihood ratio tests where age and BMI were included as continuous indicators, whereas sex and ethnicity were categorical. We tested for nonlinearity using the quadratic form of 25(OH)D and interpreted the associations based on stratified associations in the presence of curvature.

We used linear and nonlinear MR analyses to explore the causal effects of 25(OH)D on brain volumes and the risk of dementia and stroke (Supplemental Text S4). For the linear MR analyses, we used 2-sample random-effects inverse variance weight MR as the primary approach, with this method providing valid causal estimates if there is no unbalanced directional horizontal pleiotropy (35). For additional sensitivity analyses, we included weighted median (36), weighted mode (37), MR pleiotropy residual sum and outlier (MR-PRESSO) (38), and MR-Egger (39) analyses that all rely on different assumptions relating to underlying pleiotropy (**Supplemental Text S5**) (15). The 2-sample MR analyses relied on variant–25(OH)D estimates taken from the SUNLIGHT consortium (32) and variant–outcome estimates from the UK Biobank from a model adjusted for age, sex, assessment center, 40 principal components, genotyping array, and birth location. In contrast, the nonlinear MR approach used GRS in a 1-sample setting that involved the following steps: first, we generated 40 strata using “instrument-free” 25(OH)D information, reflecting 25(OH)D concentrations from which the genetic effects had been removed, obtained by taking the residual from the regression of the 25(OH)D concentration on 25(OH)D GRS (40). We then generated localized average causal effect (LACE) estimates [which is the ratio of the GRS–outcome association estimates to GRS–25(OH)D association estimates] within each stratum and conducted a meta-regression of the LACE estimates against the mean of 25(OH)D in each stratum to test whether the fractional polynomial model fits better than the linear model (40, 41). To test for the model assumptions of a uniform GRS–25(OH)D association across all 40 strata, we examined the stratum-specific associations. We found that strata 1–2 and 37–40 were outliers (**Supplemental Figure 2**), and hence, we used nonlinear MR analysis, removing these strata for our primary results. Furthermore, we repeated the linear MR analyses including the pleiotropy robust methods in models stratifying according to baseline concentration genetic instrument-free 25(OH)D (<24.9 nmol/L, 25–49.9 nmol/L, 50–74.9 nmol/L, and ≥75 nmol/L) (15, 42). We further examined possible pleiotropy using leave-block-out analysis (**Supplemental Text S6**), which involves repeating the nonlinear MR analysis using GRS by excluding SNPs in the block (functional blocks and variants included in the block found in **Supplemental Table 4**). Potential impact fraction (PIF) (43) was calculated to estimate dementia incidence that may be preventable by correction of low vitamin D status in this population using estimates obtained from the nonlinear MR analyses.

All observational analyses were conducted using the STATA SE version 14.1 software (StataCorp LLC), and the MR analyses were conducted in R version 3.6.1 (R Project for Statistical Computing) using TwoSampleMR (44), MR-PRESSO (38), and nonlinear MR packages (40).

## Results

The median follow-up for both dementia and stroke incidence was 10.9 y. The total person-years at risk were 5,467,740.9 y for dementia and 5,371,196.5 y for stroke. **Table 1** represents the distribution of the study population by baseline characteristics. Most of the participants were women aged 60–73 y at baseline without long-standing illnesses or depression and from British, Irish, or white backgrounds. 25(OH)D concentrations varied by social and lifestyle covariates, and the type of physical activity, time spent outdoors, sun protection use, oily fish consumption, and dietary restrictions were also associated with brain volumes and the incidence of dementia and stroke (**Supplemental Table 5**). Across the covariates, categories associated with lower total brain volume tended to reflect a higher rate of dementia and stroke (Supplemental Table 5).

**TABLE 1:** Summary of baseline characteristics of the UK Biobank cohort^1^

Characteristic	*n* (%)
Sex	
Male	199,275 (46.6)
Female	228,415 (53.4)
Age, y	
40–49	102,588 (24.0)
50–59	142,304 (33.3)
60–73	182,798 (42.7)
Ethnic background	
British, Irish, or white	403,475 (94.3)
Indian, Pakistani, Bangladeshi, or Asian	7709 (1.8)
African, Caribbean, or black	6793 (1.6)
Chinese	1362 (0.3)
Mixed or other ethnic groups	6366 (1.5)
Missing	1985 (0.5)
BMI, kg/m^2^	
Underweight <18.5	2196 (0.5)
Normal 18.5–25	140,027 (32.7)
Overweight 25–30	180,706 (42.3)
Obese ≥30	103,161 (24.1)
Missing	1600 (0.4)
Education	
None	71,245 (16.7)
Intermediate (NVQ/CSE/A-levels)	149,049 (34.8)
High (degree/professional)	202,414 (47.3)
Missing	4982 (1.2)
Townsend deprivation index	
Less deprived	215,258 (50.3)
Highly deprived	211,912 (49.6)
Missing	520 (0.1)
Type of physical activity	
None	26,736 (6.2)
Light/moderate	354,236 (82.8)
Strenuous sport	44,464 (10.4)
Missing	2254 (0.5)
Time spent outdoors in summer	
None	871 (0.2)
<2 h	142,051 (33.2)
3–5 h	177,648 (41.5)
>6 h	82,238 (19.2)
Missing	24,882 (5.8)
Frequency of sun protection use	
Never goes out in the sunshine	2533 (0.6)
Never/rarely	42,940 (10.0)
Sometimes	142,328 (33.3)
Most of the time	150,926 (35.3)
Always	87,612 (20.5)
Missing	1351 (0.3)
Depression	
Yes	44,394 (10.4)
No	383,296 (89.6)
Longstanding illness/infirmity/disability	
Yes	133,428 (31.2)
No	283,384 (66.3)
Missing	10,878 (2.5)

1 CSE, certificate of secondary education; NVQ, national vocational education.

### Observational analysis

Association between 25(OH)D and all neuroimaging features strengthened after adjustment for socioeconomic factors (**Table 2**). Accounting for lifestyle, sun behavior, and illness-related covariates led to some attenuation, but all associations persisted even after full adjustment (Table 2). Sensitivity analyses including further adjustment for the duration between the baseline and imaging visits or where participants with incident stroke or dementia were excluded also supported an association between 25(OH)D and all neuroimaging features (**Supplemental Figure 3**).

**TABLE 2:** The association between 25-hydroxyvitamin D (per 10 nmol/L) with brain volumes and the risk of dementia and stroke with progressive covariate adjustment^1^

Outcome	Model	β (95% CI)	*P*-trend	*P*-nonlinear^2^
Total	Basic	586.9 (230.9, 943.0)	1.2 $\times $ 10^–3^	2.8 $\times $ 10^–4^
(*N* = 31,025)	Socioeconomic	742.7 (382.9, 1102.5)	5.2 $\times $ 10^–5^	1.2 $\times $ 10^–4^
	Lifestyle	636.6 (261.4, 1011.8)	8.8 $\times $ 10^–4^	3.3 $\times $ 10^–5^
	Sun behaviors	561.8 (183.5, 940.1)	3.6 $\times $ 10^–3^	5.4 $\times $ 10^–5^
	Illness	548.0 (170.0, 925.9)	4.5 $\times $ 10^–3^	9.2 $\times $ 10^–5^
Gray matter	Basic	452.5 (234.6, 670.4)	4.7 $\times $ 10^–5^	5.0 $\times $ 10^–4^
(*N* = 31,037)	Socioeconomic	563.3 (343.4, 783.2)	5.2 $\times $ 10^–7^	2.0 $\times $ 10^–4^
	Lifestyle	403.8 (175.5, 632.1)	5.3 $\times$ 10^–4^	9.3${\rm{\ }} \times$ 10^–5^
	Sun behaviors	389.0 (158.8, 619.2)	9.3 $\times $ 10^–4^	1.2${\rm{\ }} \times $ 10^–4^
	Illness	374.5 (144.5, 604.4)	1.4 $\times $ 10^–3^	2.1${\rm{\ }} \times $ 10^–4^
White matter	Basic	134.4 (–87.0, 355.8)	0.23	0.02
(*N* = 31,037)	Socioeconomic	179.4 (–44.5, 403.2)	0.12	0.01
	Lifestyle	232.8 (–1.5, 467.1)	0.05	6.6${\rm{\ }} \times $ 10^–3^
	Sun behaviors	172.8 (–63.6, 409.2)	0.15	9.5${\rm{\ }} \times $ 10^–3^
	Illness	173.5 (–62.8, 409.8)	0.15	0.01
Hippocampal	Basic	3.1 (–2.6, 8.8)	0.29	0.04
(*N* = 31,025)	Socioeconomic	4.7 (–1.0, 10.5)	0.11	0.04
	Lifestyle	2.8 (–3.2, 8.8)	0.36	0.02
	Sun behaviors	1.6 (–4.5, 7.7)	0.61	0.03
	Illness	1.4 (–4.7, 7.5)	0.65	0.04
White matter hyperintensities^3^	Basic	–7.7 (–12.9, –2.6)	3.0 $\times $ 10^–3^	0.34
(*N* = 29,989)	Socioeconomic	–11.9 (–17.1, –6.7)	6.4 $\times $ 10^–6^	0.27
	Lifestyle	–5.2 (–10.6, 2.0)	0.06	0.40
	Sun behaviors	–6.5 (–11.9, –1.1)	0.02	0.37
	Illness	–6.1 (–11.5, –7.0)	0.03	0.55
		HR (95% CI)		
Dementia	Basic	0.86 (0.83, 0.88)	2.7 $\times $ 10^–23^	5.6 $\times {\rm{\ }}10$^–22^
(*N* = 372,232)	Socioeconomic	0.84 (0.82, 0.87)	3.4$\ \times $ 10^–27^	5.7 $\times {\rm{\ }}10$^–19^
	Lifestyle	0.85 (0.82, 0.88)	6.0 $\times $ 10^–23^	1.4${\rm{\ }} \times {\rm{\ }}10$^–15^
	Sun behaviors	0.86 (0.83, 0.88)	4.2 $\times $ 10^–21^	8.5 $\times {\rm{\ }}10$^–14^
	Illnesses	0.88 (0.85, 0.9)	3.4 $\times $ 10^–16^	2.1 $\times {\rm{\ }}10$^–9^
Stroke	Basic	0.93 (0.90, 0.95)	3.4 $\times $ 10^–11^	9.7 $\times {\rm{\ }}10$^–7^
(*N* = 372,232)	Socioeconomic	0.92 (0.90, 0.95)	1.6 $\times $ 10^–11^	2.9 $\times {\rm{\ }}10$^–5^
	Lifestyle	0.94 (0.92, 0.97)	1.9 $\times $ 10^–6^	8.7 $\times {\rm{\ }}10$^–4^
	Sun behaviors	0.94 (0.92, 0.97)	1.4 $\times $ 10^–6^	1.3 $\times {\rm{\ }}10$^–3^
	Illnesses	0.95 (0.93, 0.97)	3.8 $\times $ 10^–5^	0.01

1 Estimates for brain volumes from linear regression, risks of dementia, and stroke assessed using Cox proportional hazards models. Adjustments were as follows: basic covariates (model 1): age, sex, assessment center, ethnicity, month; socioeconomic (model 2): model 1 + education, Townsend deprivation index, employment status; lifestyle factors (model 3): model 2 + BMI (categorical), type of physical activity, diet quality, any use of dietary supplements; sun behaviors (model 4): model 3 + time spent outdoors in summer, time spent outdoors in winter, sun protection; illnesses (all covariates): model 4 + long-standing illnesses/disability/infirmity + depression.

2 *P*-nonlinear from a model using a quadratic term of 25-hydroxyvitamin D in nmol/L.

3 White matter hyperintensitiy volume log-transformed in analyses due to skewness.

**FIGURE 2:**
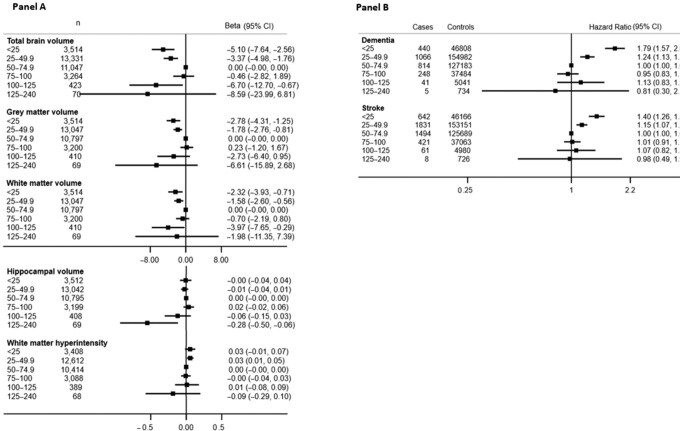
The adjusted observational associations between categories of 25-hydroxyvitamin D [25(OH)D] with neuroimaging outcomes (A) and disease outcomes (B). Participants with 25(OH)D 50–74.9 nmol/L are used as reference. Estimates obtained from linear regression for neuroimaging outcomes and from Cox proportional hazards model for disease outcomes. All models are adjusted for basic covariates (age, sex, assessment center, ethnicity, month), socioeconomic factors (education, Townsend deprivation index, employment status), lifestyle factors [BMI (categorical), type of physical activity, diet quality, any use of dietary supplements], sun behaviors (time spent outdoors in summer, time spent outdoors in winter, sun protection), and disease outcomes (long-standing illnesses/disability/infirmity, depression).

The association between 25(OH)D and total brain, gray matter, white matter, and hippocampal volume was nonlinear (*P*-nonlinear < 0.04 for all) (Table 2). For total brain, gray matter, and white matter volume, the association with 25(OH)D concentrations was U-shaped with both low and high 25(OH)D concentrations associated with lower brain volumes, whereas for hippocampal volume, the association was apparent only in the upper extreme (**Figure 2**, **Supplemental Figure 4**). There was also some evidence for an association between lower 25(OH)D concentrations and white matter hyperintensity volume (25–49.9 nmol/L compared with 50–74.9 nmol/L; adjusted β: 0.03; 95% CI: 0.01, 0.05) (Figure 2A). There was some heterogeneity in the strength of the association between 25(OH)D and total brain, gray matter, and white matter volume between males and females (*P*-interaction < 0.01 for all), and although the coefficients were generally in the same direction, associations appeared stronger for males than for females (**Supplemental Table 6**). No evidence for interaction was observed by age, ethnicity, or BMI in the association of 25(OH)D concentrations with any of the neuroimaging features.

There was an association between 25(OH)D and dementia and stroke risk that persisted after full adjustment (Table 2). These associations were nonlinear (adjusted *P*-nonlinear = 2.1 $\times {\rm{\ }}10$^–9^ and 0.01, respectively), but we did not observe any evidence for effect modification by age, sex, ethnicity, or BMI (*P*-interaction > 0.05 for all comparisons). The highest risks of both dementia and stroke were seen for participants with the lowest concentrations (<50 nmol/L) of 25(OH)D, with no differences in risk by increasing concentrations (Figure 2B, **Supplemental Figure 5**).

### Mendelian randomization

25(OH)D concentrations were instrumented by GRS including 35 variants, which jointly explained 2.8% of the variation in the UK biobank (*F*-statistic = 8,672, *P* < 1.0$\ \times \ {10^{ - 300}}$). 25(OH)D GRS was not associated with confounders (uncorrected *P* > 0.09 for all, **Supplemental Table 7**).

**FIGURE 3:**
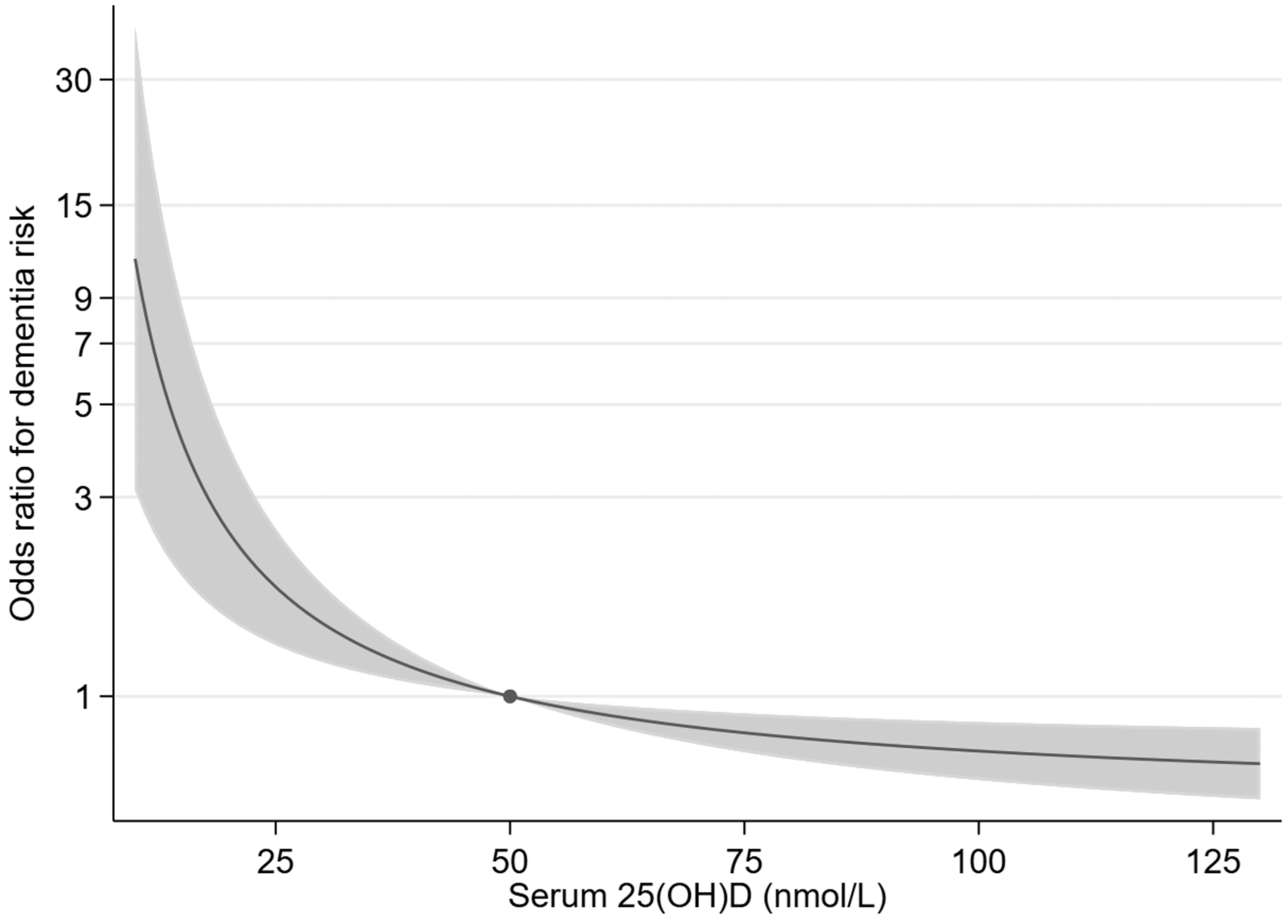
The association between genetically determined 25-hydroxyvitamin D [25(OH)D] and odds of dementia using a fractional polynomial model with the outlying strata removed (*P*-nonlinearity = $5.9\ \times {10^{ - 3}}$ for this association). The dot indicates the reference point at 50 nmol/L, and the shaded areas represent the 95% CIs.

In linear MR analyses, 25(OH)D was not associated with any of the neuroimaging outcomes or the risk of dementia or stroke (**Supplemental Table 8**, *P* > 0.10 for all linear associations). There was no evidence for pleiotropy (MR–Egger *P*-intercept > 0.90 for all associations), and the MR-PRESSO outlier test did not detect evidence for pleiotropic variants. However, we found evidence for a nonlinear inverse association between genetically determined 25(OH)D and dementia risk, with the odds of dementia decreasing with higher 25(OH)D concentrations until ∼50 nmol/L (**Figure 3**, **Supplemental Figure 6**). Based on the nonlinear MR, individuals with serum 25(OH)D at 25 nmol/L had 54% (95% CI: 1.21, 1.96) higher odds of dementia compared with those with 50 nmol/L. The shape of the polynomial model was affected by inclusion of variants in the “blood traits functional block,” with further investigation using leave-one-out analysis showing that the shape of the fractional polynomial was sensitive to the inclusion of the *GC* variant to the GRS (**Supplemental Table 9**). In stratified MR analyses, higher 25(OH)D was associated with lower odds of dementia among those with the lowest concentrations (<25 nmol/L, inverse variance weighted MR OR: 0.35; 95% CI: 0.19, 0.63 per 10 nmol/L higher), with no evidence for pleiotropy and consistent findings across all MR approaches (**Supplemental Figure 7**). There was no consistent evidence for lower dementia risk by higher 25(OH)D for individuals with concentrations >25 nmol/L (Supplemental Figure 7). According to PIF calculation, up to 17% (95% CI: 7.22, 30.58) of dementia could be prevented in this population by increasing the serum 25(OH)D to 50 nmol/L for those with a serum value below this threshold (**Supplemental Figure 8**).

## Discussion

Our analyses using a large cohort of UK participants provides some evidence for a beneficial role for adequate 25(OH)D concentrations on brain health. In the observational analyses, low 25(OH)D concentration was associated with differences in several aspects of brain morphometry, in addition to increased risks of dementia and stroke. Although we were not able to confirm causality concerning the effects on brain volumes or the risk of stroke, we did observe evidence for a nonlinear causal association between 25(OH)D and dementia risk in the MR analyses. This suggests that the benefit of improving 25(OH)D concentrations is likely to be strongest and may be restricted to the context of alleviating vitamin D deficiency, and attempts to increase concentrations beyond 50 nmol/L may provide limited further benefit. These findings highlight the importance of treating and preventing vitamin D deficiency, as well as appreciating the challenges to obtain evidence from RCTs, which may be deemed unethical or infeasible to confirm the causal effects of supplementation in those with very low 25(OH)D concentrations (15).

Findings on the association between 25(OH)D and brain volume have been largely based on cross-sectional studies, which cannot explore temporal associations. Consistent with a meta-analysis conducted in 2014, we found that lower 25(OH)D is associated with lower total brain volume (7). Furthermore, in line with a recent cross-sectional study (45), our study supports a threshold effect, where both lower and higher 25(OH)D concentrations are associated with lower total brain, gray matter, and white matter volumes, all of which are markers reflecting brain atrophy and an elevated risk of cognitive decline and dementia. However, in our study, vitamin D deficiency was not associated with lower hippocampal volume, which is a key prognostic marker for dementia risk, and we observed only an association with high concentrations (>125 nmol/L). This contrasts with at least 2 cross-sectional studies (11, 12) and 1 prospective study (46), which have all reported an association between vitamin D deficiency and lower hippocampal volumes. We did observe an association between low 25(OH)D and greater white matter hyperintensity volume, which is interesting as a greater prevalence of these lesions in the brain could suggest increases in dementia risk (47). Indeed, an association between low 25(OH)D and white matter hyperintensity volume has been consistently supported by earlier cross-sectional studies in institutionalized and hospitalized elderly participants, in whom also vitamin D deficiency is relatively common (8–10). However, to our knowledge, only 1 earlier prospective study (*N* = 1658), which was conducted on a general population sample, did not find evidence for an association (46). Although studies on the association between 25(OH)D concentrations and neuroimaging features appear to suggest a potential role in brain morphology, further well-designed prospective studies are required to clarify these associations and possible threshold effects.

The finding that the strongest effects on dementia risk are seen for those with the lowest 25(OH)D concentrations in our study was consistent in both observational and MR analyses. Evidence for a threshold effect has also been observed in other studies, including a meta-analysis of 5 prospective studies that found a pooled 33% and 14% higher risk of dementia in vitamin D–deficient (<30 nmol/L) and vitamin D–insufficient (30–50 nmol/L) participants, respectively, compared with those with sufficient concentrations (>50 nmol/L) (13). Evidence from RCTs using vitamin D supplementation to prevent dementia or stroke is limited, and these studies have typically been small, have been of short duration, and may not have included participants with overt deficiency (2). Although other prospective studies have reported a similar U-shaped association with the risk of stroke as seen in our study (14), to our knowledge, evidence for a causal association between 25(OH)D and stroke risk has not been reported by any of the MR studies conducted to date (19, 20). In contrast, 3 linear MR studies support a causal association between 25(OH)D and Alzheimer disease (16–18). Our nonlinear MR analyses suggested a threshold effect, in which increases in 25(OH)D would mainly benefit individuals who have an overt vitamin D deficiency. Interestingly, in our study and in the previous linear MR studies (16–18), the association between 25(OH)D and dementia appears to be sensitive to the removal of *GC* variants. *GC* encodes the vitamin D binding protein, and it is the strongest individual variant affecting 25(OH)D concentrations. As in our study, the association between 25(OH)D and dementia within the deficiency threshold was consistent across all MR methods, and no influential variants were observed; the apparent sensitivity to *GC* may reflect lack of power in the analyses excluding this variant.

A protective effect of higher 25(OH)D on brain health is biologically plausible and could be explained by at least 3 potential mechanisms. First, the presence of vitamin D receptors in the hypothalamus has suggested a neurosteroid function for active vitamin D, promoting the growth and maturation of neurons (48, 49). Second, there may be vascular mechanisms as active vitamin D has been associated with reduced thrombosis and regulation of the renin–angiotensin system (5). Third, replete concentrations of active vitamin D may act as a neuroprotectant through the suppression of excess inflammatory neurovascular damage caused by proinflammatory cytokines and attenuation of amyloid proteins, commonly observed in Alzheimer disease (3, 4).

There are several strengths to our study. First, as one of the largest population-based prospective and MR studies investigating the association between 25(OH)D and brain health, our study had more statistical power to detect associations than past smaller studies. Second, the extensive data available allowed for comprehensive adjustments of confounders and enabled the investigation of effects at very low concentrations of 25(OH)D. Third, due to the nature of MR studies, the findings from our MR study are less influenced by reverse causality and confounding (15), with our results being robust across several sensitivity analyses, including pleiotropy robust methods. Finally, to our knowledge, this is the first study to conduct nonlinear MR analyses and to provide causal evidence for a role of 25(OH)D for which dementia risk appears to operate only below the deficiency threshold.

There were also several limitations to our study. Although we used extensive adjustment strategies, we cannot rule out influences by residual confounding in our observational analyses. In addition, although the UK Biobank cohort is diverse, it is vulnerable to healthy volunteer bias as the baseline population was mostly less deprived, had intermediate to high education, and had a normal to overweight BMI (50). As the MR analyses were restricted to participants from white British ancestry, these findings may not be generalizable to other populations. Although the genetic instruments included in our analyses all had a replicated associations with 25(OH)D concentrations, we cannot fully exclude the possibility of horizontal pleiotropy in which the genetic variant of interest affects the outcome through another phonotype or pathway (15). In particular, the possible independent role of vitamin D binding protein (*GC*) requires further scrutiny, even if extensive sensitivity analyses using multiple MR approaches did not find evidence for pleiotropy. In addition, the subsample available for brain morphometry analyses may have been too small to detect a difference in the MR analyses, despite us using the largest study available. For instance, in contrast to the 23,901 participants with MR information, the sample would need to be increased 10-fold (*N* = ∼279,893) to provide 80% power for detecting the type of difference in total brain volumes seen in our study at the 5% level. Furthermore, given differences in 25(OH)D concentrations measured by different assays and the small number of individuals with 25(OH)D >100 nmol/L in our study, it is not possible to infer potential effects of high concentrations. It might also be argued that as residual concentrations (where the effect of genetic variation was removed) were used to establish thresholds for 25(OH)D in the nonlinear MR analyses, it is unclear how those relate to measured concentrations. However, given the correlation between residual and measured 25(OH)D concentrations is very strong (*r* = 0.986), any difference is likely to be trivial.

In conclusion, our study supports a role of vitamin D deficiency on brain health, notably for the risk of dementia. Larger MR studies are needed to confirm causality for the proposed associations between 25(OH)D concentrations and brain morphometry. Our MR results suggest no clear association with stroke, whereas a causal relation with dementia risk provides an important opportunity for prevention.

## Supplementary Material

nqac107_Supplemental_File

## Data Availability

Data described in the manuscript, code book, and analytic code will be made available upon request pending application to the UK Biobank.

## Notes

Supported by the National Health and Medical Research Council under grants GNT1157281 and GNT1123603.

Supplemental Tables 1–9, Supplemental Figures 1–8, and Supplemental Text S1–S6 are available from the “Supplementary data” link in the online posting of the article at https://academic.oup.com/ajcn/.

Abbreviations: FLAIR, fluid-attenuated inversion recovery; GRS, genetic risk score; GWAS, genome-wide association study; LACE, localized average causal effect; MR, Mendelian randomization; MR-PRESSO, Mendelian randomization pleiotropy residual sum and outlier; PIF, potential impact fraction; RCT, randomized controlled trial; SNP, single-nucleotide polymorphism; SUNLIGHT, Study of Underlying Genetic Determinants of Vitamin D and Highly Related Traits; TE, time of echo; TR, time of repetition; 25(OH)D, 25-hydroxyvitamin D.
