# Supplementary material for: Vitamin D and brain health: an observational and Mendelian randomization study
Source: Am J Clin Nutr. 2022 Apr 22;116(2):531–40. doi: 10.1093/ajcn/nqac107 (PMC9348994; doi:10.1093/ajcn/nqac107)
Supplement: nqac107_Supplemental_File [file nqac107_supplemental_file.docx]

**Vitamind D and brain health: an observational and Mendelian randomisation study**

Shreeya S Navale (BMedRadSc)(Hons), Anwar Mulugeta (PhD), Ang Zhou (PhD), David J Llewellyn (PhD)^,^ and Elina Hyppönen (PhD)

Australian Centre for Precision Health, Unit of Clinical and Health Sciences, University of South Australia, Adelaide, Australia (SSN, AM, AZ, EH)

South Australian Health and Medical Research Institute, Adelaide, Australia (AM, AZ, EH)

Department of Pharmacology and Clinical Pharmacy, College of Health Science, Addis Ababa University, Addis, Ababa, Ethiopia (AM)

College of Medicine and Health, University of Exeter, Devon, England (DJL)

Alan Turing Institute, London, England (DJL)

**Corresponding Author:**

Professor Elina Hyppönen, Australian Centre for Precision Health, University of South Australia, South Australian Health & Medical Research Institute (SAHMRI) Level 8, GPO Box 2471, Adelaide SA 5001, Australia.
Tel: +61 8 8302 2518
Email: [elina.hypponen@unisa.edu.au](mailto:elina.hypponen@unisa.edu.au)

**Table of contents**

**Supplementary Text** ……………………………………………………………..5

**Supplementary Table 1:** Neuroimaging outcomes and their link with different types of dementias……………………………………………………………….11

**Supplementary Table 2:** Table with definition, categorisation, and method of data collection of all covariates used in the study ……………………………………………12

**Supplementary Table 3:** Vitamin D variants used for the genetic instruments for serum 25(OH)D concentrations and their Genome wide association…………………15

**Supplementary Table 4.** Functional blocks used in the leave-block-out analyses…..18

**Supplementary Table 5:** Summary of 25(OH)D, Total Brain Volume (TBV), White Matter Hyperintensity volume (WMH), the incidence of dementia and stroke, by baseline characteristics of the UK Biobank cohort……………………………………..19

**Supplementary Table 6:** The association between 25(OH)D (per 10 nmol/L) with neuroimaging outcomes and the risk of dementia and stroke with progressive covariate adjustment for males and females…………………………………………….21

**Supplementary Table 7**: Vitamin D genetic risk score (Vitamin D-GRS) association with potential confounders in the UK biobank …………………………………….22

**Supplementary Table 8:** The association between genetically determined 25(OH)D and neuroimaging outcomes and risk of dementia and stroke, using Two Sample MR and non-linear MR analyses…………………………………………………………….23

**Supplementary Table 9:** Leave-block-out analysis for non-linear MR analysis of serum 25(OH)D on dementia risk…………………………………………………..24

**Supplementary Figure 1:** Flow diagram showing the analytical sample size for the observational and mendelian randomisation analysis (diagram in dotted box) involving total and regional brain volumes, dementia, and stroke outcomes………25

**Supplementary Figure 2:** The estimates for the association between 25(OH)D and genetic score (vitamin D-GRS-25(OH)D) across the 40 residual 25(OH)D-based strata.….26

**Supplementary Figure 3**: The observational association of 25(OH)D with brain imaging outcomes for the full model in the main analysis (main result), and three scenarios of sensitivity analysis. ………………………………………………………………27

**Supplementary Figure 4:** Association between categories of 25(OH)D and TBV (panel A), GMV (panel B), WMV (panel C), HV (panel D) and WMH (panel E), using linear regression, with 50-74.9nmol/L of 25(OH)D as reference……………………..28

**Supplementary Figure 5:** Association between categories of 25(OH)D and risk of dementia (panel A) and stroke (panel B), using cox proportional hazards model, with 50-74.9nmol/L of 25(OH)D as reference………………………………………………..31

**Supplementary Figure 6:** Association between genetically determined 25(OH)D and odds ratio of dementia using a fractional polynomial model before removing outlying data………………………………………………………………………………33

**Supplementary Figure 7:** Overall unstratified and stratified causal linear associations between 25(OH)D and risk of dementia using IVW, MR-PRESSO, Weighted median, Weighted mode, MR-Egger methods of TwoSample MR analysis………………………………………………………………………………..34

**Supplementary Figure 8:** The potential impact of increasing serum 25(OH)D to different thresholds on dementia risk reduction………………………………………35

**Supplementary text**

**Text S1: Handling related individuals**

UK Biobank genetic analysis team identified genetic relatedness for around 16% (~81,000) UK biobank participants. To minimise the bias due to relatedness and to maximize the sample size for our analysis, we applied two strategies one for the observational analysis and one for Mendelian randomisation analysis. Given the neuroimaging data was available only for subsample of the UK Biobank, to maximize the sample size for the observational analysis we included up to two participants per “family”, using models weighted by 1-kinship coefficient (1) For MR analyses, we restricted the analysis to unrelated individuals to minimise the bias due to population structure that may be introduced by inclusion of related individuals (2).

**Text S2: Serum 25-hydroxyvitamin D (25(OH)D) assays**

Serum 25(OH)D concentration was determined from samples collected at baseline using direct competitive chemiluminescent immunoassay (DiaSorin Liaison XL), with the assay having a measuring range of 10-375nmolL^-1^. 25(OH)D values below (n=2,654) or above (n=2) the reportable limit were replaced with missing (3). Furthermore, given the dilution issues identified during the quality checks more so from aliquot 3, we excluded participants (n=10,002) with 25(OH)D data from aliquot 3 (Supplementary Figure 1). Details on the assay analyzer performance and quality control of serum biomarkers including 25(OH)D is show below (3, 4). The assay was registered with External Quality Assurance sheme (RIQAS Immunoassay Speciality 1), with all distirbutions assessed as Good or Acceptable (1).

**Serum assay performance characteristics**

| **IQC level** | **External IQC material range** | **Average within-Laboratory (total) CV%** | **Average SD** | **Comment ^1^** |
| --- | --- | --- | --- | --- |
| Low | 26.0-49.9 | 6.14 | 2.21 | 2 QC types- 4 different lots9 |
| Medium | 54.2 – 85.9 | 5.39 | 3.81 | 2 QC types- 4 different lots9 |
| High | 78.1-116 | 5.04 | 4.73 | 2 QC types- 4 different lots9 |

^1^Initially 3 levels of Randox IQC material were used but due to difficulties receiving IQC material in time, there were some periods where all 3 levels could not be analysed. To continue analysis a decision was made to analyse 3 levels of Technopath IA IQC material along with the available level(s) of Randox IQC material. As soon as all 3 levels of Randox IQC were available this practice stopped.

**Text S3: Brain MRI in UK Biobank**

**Pre-processing neuroimaging data**: All data was converted from a digital imaging and communications in medicine (DICOM) to a network interface to file transfer in the internet (NIFTI) format (5). Then, unusable data, which was defined as any raw imaging data with wrong dimensions, corrupted data, missing or incompatible images, were identified and removed (6). From 2014 to 2016, 98% of the T1-weighted images and 83% of T2-FLAIR images were deemed “usable” (6). The data was then organized by each modality (T1-weighted or T2-FLAIR) and anonymized by removing any sensitive header information that may help identify the participants (5).

**Quality control**: Further automated quality control processes were implemented to check for 190 possible problems (6). Some common problems included excessive head motion in images, discrepancy between the T1-weighted image and the standard template (population average) due to alignment issues or corrupted data, discrepancy between T1-weighted image and the other modalities after alignment, signal to noise ratio and contrast to noise ratio (5, 6). Images identified with serious problems were removed (6).

**Automated processing pipeline T1:** The images were defaced, a gradient distortion correction was performed and the field of view was reduced to minimize non-brain tissue above and below the brain area (5). The original image was transformed into a standard (population average) template using FMRIB’s linear image registration tool (FLIRT) and FMRIB’s nonlinear image registration tool (FNIRT) and then, a brain extracted T1-weighted image is produced (5). Structural image evaluation using normalization of atrophy cross-sectional (SEINAX)-style analysis was used to estimate the external surface of the skull. This estimate forms the head scaling factor which is used to normalize brain volumes in our analysis (5, 6). Finally, the brain image is segmented into different tissues-types and subcortical structures using FMRIB’s automated segmentation tool (5).

**Automated processing pipeline T2-FLAIR:** Images were defaced, gradient distortion correction was applied and then the T2-FLAIR images are aligned to T1-weighted images using FLIRT to subsequently convert them to standard space (5, 6). Through the combined data from T1-weighted and T2-FLAIR images, lesions are automatically segmented using the Brain intensity Abnormality Classification Algorithm (5).

**Text S4: Genetic risk score**

We constructed genetic risk scores (GRS, see the equation below) using 35 common single nucleotide polymorphisms that were identified in a GWAs on 25(OH)D using information from 417,580 European ancestry individuals from UK Biobank (7), and which had at least a nominally significant and directionally consistent association with 25(OH)D in the earlier independent GWAS by the SUNLIGHT Consortium, which did not include UK Biobank (8). We multiplied the number of 25(OH)D increasing alleles in each SNP by the variant-25(OH)D association estimates (beta coefficients) from SUNLIGHT consortium (8) and then summed the weighted alleles (EQUATION 1) Supplementary Table 2). Finally, the score was rescaled by the ratio of the available number of variants to the sum of beta coefficients to express the association estimates per 25(OH)D increasing alleles.

EQUATION 1:

| Weighted genetic risk score (GRS) = | (β_1_ x SNP_1_ + β_2_ x SNP_2_ + … β_35_ x SNP_35_) x N_SNPs_ |
| --- | --- |
|  | Sum of β coefficients |
|  |  |
| Where: β_1_ to β_35_ is a coefficient from SNP-25(OH)D association estimates from SUNLIGHT consortium, SNP1 to SNP35 are the number of 25(OH)D concentration increasing alleles for each SNP and N_SNPs_ is the available number of SNPs per individuals. | |

**Text S5:Two-sample MR analysis**

We first investigated the linear relationship of 25(OH)D with brain volumes and the risk of dementia and stroke. Upon evidence of non-linear causal relationship from fractional polynomial MR analysis, we carried out further linear MR analyses based on groups stratified by instrument-free 25(OH)D concentration (≤25nmol/L, 25-50nmol/L, 50-75nmol/L and ≥75nmol/L). Instrument free (instead of “raw” 25(OH)D) was used as stratification by the raw 25(OH)D concentrations could introduce a collider bias (9). Instrument-free 25(OH)D concentration was generated for each individual by substracting the centred genetic contribution of the GRS to the 25(OH)D levels from the total serum 25(OH)D concentration (“raw” 25(OH)D concetration) (10). In both two sample-MR scenarios, we used random-effects inverse variance weight (IVW) MR as the primary approach (11), with additional analyses conducted using pleiotropy robust MR methods including weighted median (12), weighted mode (13), MR-Egger (14) and MR-PRESSO (15). IVW MR is a regression of variant-exposure association estimates against the estimate of variant-outcome association weighted for the inverse variance of the variant-outcome association, with the intercept constrained to zero (11). To account for balanced horizontal pleiotropy (where the pleiotropic effects of individual variants are summed to zero) we selected random-effects IVW model (11). MR-Egger analyses allows unbalanced horizontal pleiotropic effects given the intercept for variant-exposure against variant-outcome regression is not constrained to zero (14). However MR-Egger assumes that the pleiotropic effects are independent of the variant-exposure effects (known as the InSIDE assumption). Weighted median method is robust to unbalanced horizontal pleiotropy, and does not rely on the InSIDE assumption, but it requires that the set of variants contributing 50% or more of the total weight are valid (12). Weighted modal MR calculates the estimate based on the largest cluster of variants with similar causal estimates.(13) The majority of the variants could be invalid, but providing that all the variants in the largest homogenous cluster are valid, the weighted mode estimate is asymptotically unbiased.(13) For testing the pleiotropy, we used the MR-Egger intercept test, where the significant deviation of the intercept from zero could reflect the presence of unbalanced horizontal pleiotropy (14). Outlying variants and their distorting effect on the causal estimates were also investigated using the MR-PRESSO approach (15). MR-PRESSO outlier test requires the InSIDE assumption to be valide and at least 50% of the genetic variants to be a valid instruments.

**Text S6: Leave-block-out analysis**

As the fractional polynomia model uses a GRS rather than a SNP based approach, to further account the horizontal pleiotropy, we performed leave-block-out analyses which involved subgrouping of potential pleiotropic variants into functionally related blocks (Supplementary Table 4). Here, we assumed that any SNP with an association with any other trait at P-value (p<5x10-08) in PhenoScanner search (16) is potentially pleiotropic. After a search and classificiation of SNP associations with other traits into blocks based on their potential function, we generated genetic scores excluding each block, and repeated non-linear MR analyses for each of the restriceted risk scores. Among the 35 variants, 20 SNPs were associated with at least one trait at genome-wide significant threshold (p<5x10-08) in PhenoScanner (16). We created four functional blocks including “blood traits”, “metabolic traits”, “renal traits”, and “unclassified” (variants whose associated traits did not fall into other blocks). More detail of this processed used in our recently published work (17). Of note, the functional block of ‘blood traits’ includes *GC* (rs1352846) due to a previously reported association with white cell and granulocyte count (Supplementary Table 4). *GC* is also the strongest individual 25(OH)D variant and it explains 1.6% of the variation in concentrations (from the total of 2.8%). We observed that the shape of the fractional polynomial association was sensitive to inclusion of variants in the “blood trait” functional block. We then investigated the contribution by exclusion of individual variants within the block, confirming that differences in the shape of the fractional polynomial model were driven by the GC variant (Supplementary Table 9).

**Suppplementary Table 1:** Neuroimaging outcomes and their link with different types of dementias

| Neuroimaging outcome | Definition/mechanism | Cognitive decline/commonly associated dementia |
| --- | --- | --- |
| Total brain volume | Total brain volume is the sum of white matter and grey matter volumes. Lower total brain volumes have been associated with a higher risk of cognitive decline in general (18). Total brain atrophy reflects the pathological processes in Alzheimers disease (AD) including cognitive decline and neurodegeneration arising from amyloid plaques, neurofibrillary tangles and inflammatory damage to neurons (19). | AD, lewy body dementia, mild and subjective cognitive decline, frontotemporal dementia, vascular dementia (20) |
| Grey matter volume | Grey matter volume is composed of cell bodies. Grey matter atrophy is a normal sign of ageing. However, lower grey matter volumes have been associated with cognitive decline as measured by MMSE scores especially for people with prodromal AD and AD. (21) | AD, subjective and mild cogntivie impairment, lewy body dementia, frontotemporal dementia (20) |
| White matter volume | White matter consists of areas of the brain which include myelin axons and various glial cells which play a vital role in maintaining the BBB, axons and myelin (22). White matter atrophy can indicate strural integrity loss due to demyelination and death of axonal processes. It can also indicate an expansion of cerebro spinal fluid spaces (19). | Vascular dementias,AD (20, 22) |
| Hippocmapal volume | A region of the brain in the temporal lobe. This is one of the first areas which atrophies develop in AD and is often considered a biomarker of AD. Amyloid PET neuroimaging suggests that it may be due to increased amyloid protein deposition, which is often formed in the pathogenesis of AD (18). It is also functionally associated with one of the early symptoms of AD which is memory impairment (19). | AD, subjective and mild cognitive decline,lewy body dementia (20) |
| White matter hyperintensities | Areas of the white matter at which there is cerebrovascular compromise resulting in alterations in normal white matter. Often associated with aging process. However, a greater volume has been associated with cognitive decline, stroke and dementia (22). | AD, vascular dementias and vascular related cognitive decline, executing and processing cognitive declines, frontotemporal dementia (20, 22) |

**Supplementary Table 2:** Table with definition, categorisation, and method of data collection of all covariates used in the study

| Covariate | Definition | Categorisation | Method of data collection |
| --- | --- | --- | --- |
| Self-reported sex | Self-reported sex of participant | Male; female | Collected from central registry and updated at recruitment |
| Genetic sex | Genetic sex of the participant | Male; female | Obtained through genotypic analysis |
| Age | Refers to the age of the participant when they attended the initial assessment (age at recruitment) | 40-49 years; 50-59 years; 60-73 years | Calculated through the date of birth and date of attending the assessment centre |
| Assessment centre | The assessment centre at which the participant had consented | Barts; Birmingham; Bristol; Bury; Cardiff; Cheadle; Croydon;  Edinburgh; Glasgow; Hounslow; Leeds; Liverpool; Manchester  Middlesbrough: Newcastle: Nottingham: Oxford: Reading;  Sheffield; Stockport; Stoke; Swansea; Wrexham; Cheadle  (imaging); Reading (imaging); Newcastle (imaging) | Recorded automatically when participant consented |
| Monthly variations | The month the participant attended the assessment centre on baseline visit (2006-2010) and first repeat visit (2012-13) | January to December | Automatically acquired at reception during blood collection |
| Ethnic group | Ethnic group of the participant | British, Irish or White; Indian, Pakistani, Bangladeshi or Asian; African, Caribbean or Black;  Chinese; Mixed or other ethnic groups | Through touchscreen questionnaires, participants were asked the following questions:  “What is your ethnic group?” where they chose from the following options:  “(1) White, (2) Mixed, (3) Asian or Asian British, (4) Black or Black British, (5) Chinese, (6) Other ethnic group. Based on their answer, participants were asked the same questions again with options specific to their ethnic group. For example, people who answered White were asked to choose from the following options: “(1) British, (2) Irish or (3) Any other white background” |
| Skin colour | The self-reported skin colour of the participant | Very fair; Fair; Light olive; Dark olive/brown/black | Through a touchscreen questionnaire, participants were asked: "What best describes the colour of your skin without tanning?" |
| Education | Highest self-reported qualification of the participant at the time of attending the assessment centre. | None; Intermediate (NVQ/CSE/A-levels); High (degree/professional) | Through touchscreen questionnaires, participants were asked the following questions:  “Which of the following qualifications do you have?” |
| Employment status | The employment statuses of the participant at the time of employment | Paid employment or self-employed; Retired; Unemployed | Through touchscreen questionnaires, participants were asked the following questions:  “Which of the following describes your current situation?”. |
| Type of physical activity | Type of physical activity in which the participant has spent more than 10 minutes engaging in the past 4 weeks. | None; Light/moderate; Strenuous sport | Combined through answers from the following touchscreen questions: “In a typical WEEK, on how many days did you walk for at least 10 minutes at a time? (Include walking that you do at work, travelling to and from work, and for sport or leisure)”, “How many minutes did you usually spend walking on a typical DAY?”, “In a typical WEEK, on how many days did you do 10 minutes or more of moderate physical activities like carrying light loads, cycling at a normal pace? (Do not include walking)”, “How many minutes did you usually spend doing moderate activities on a typical DAY?”, “In a typical WEEK, how many days did you do 10 minutes or more of vigorous physical activity? (These are activities that make you sweat or breathe hard such as fast cycling, aerobics, heavy lifting)” |
| Time spent outdoors in winter and time spent outdoors in summer | The total number of hours spent outdoors in summer and winter by the participant. | None; <2 hours; 3-5 hours; >6 hours | Through asking touchscreen questions: “In a typical DAY in summer, how many hours do you spend outdoors?” and “In a typical DAY in winter, how many hours do you spend outdoors?” |
| Frequency of sun protection | The self-reported frequency of sun protection | Never goes out in the sunshine; Never/rarely; Sometimes; Most of the time; Always | Through the touchscreen question: “Do you wear sun protection (e.g., sunscreen lotion, hat) when you spend time outdoors in the summer?” |
| Frequency of oily fish consumption and non-oily fish consumption | The self-reported frequency of oily and non-oily fish consumption | Never; Less than once a week; Once a week; Greater than once a week | Obtained from touchscreen questions: “How often do you eat oily fish? (e.g. sardines, salmon, mackerel, herring)” and “How often do you eat other types of fish? (e.g. cod, tinned tuna, haddock)” |
| Diet restrictions | Dietary restrictions of the participant. | No eggs of dairy containing food; No wheat-containing food; No sugar, sugar-containing food or drinks; Eat all of the above | Through touchscreen question: “Which of the following do you NEVER eat?” |
| Depression | Self-reported depression based on a variety of questions | Yes; no | Self-reported depression was collected through a series of questions about depression in the depression series of questions in the online “thoughts and feelings” mental health questionnaire of the UK Biobank. |
| Diet quality | The variation in diet based on frequency of food consumption | The scale of 0-4 based on a method described in a study (23) | Through touchscreen questions asking about frequency of food intakes of meat, poultry, beef, lamb, pork, dried fruits, cooked and uncooked vegetables. |
| Any vitamin or mineral supplements | Use of any vitamins or minerals at all by the participant. | Yes; No | Through answers to touchscreen questions "Do you regularly take any of the following? (You can select more than one answer)" with an option to choose multiple vitamins and minerals or ‘none of the above’. |
| Longstanding illness/infirmity /disability | Any long-standing illnesses, disability or infirmity reported by the participant. | Yes; No | Through the touchscreen question "Do you have any long-standing illness, disability or infirmity?" |

Information obtained from Pham et al. (24) and UK biobank data showcase (25). More information can be found in the UK biobank data showcase.

**Supplementary Table 3:** Vitamin D variants used for the genetic instruments for serum 25(OH)D concentrations and their Genome wide association estimates from UK Biobank and SUNLIGHT Consortium.

|  |  |  |  |  |  |  |  | UK Biobank^1^ | | | SUNLIGHT Consortium^2^ | | | SNPs for |
| --- | --- | --- | --- | --- | --- | --- | --- | --- | --- | --- | --- | --- | --- | --- |
| No | **SNP** | **CHR** | **BP** | **Gene** | **A1** | **A2** | **A1F** | **Beta** | **SE** | **P** | **Beta** | **SE** | **P** | **vitaminD-GRS** |
| 1 | rs6671730^3^ | 1 | 2339139 | *PEX10* | G | A | 0.565714 | 0.0147881 | 0.00201077 | 1.92E-13 | 0.00610 | 0.00230 | 6.65E-03 | Yes |
| 2 | rs35408430 | 1 | 17560195 | *PADI1* | C | T | 0.657806 | 0.0214952 | 0.00209979 | 1.36E-24 | 0.02370 | 0.00565 | 2.71E-05 | Yes |
| 3 | rs7522116 | 1 | 41835685 | *FOXO6* | C | T | 0.433767 | 0.0134641 | 0.00202533 | 2.97E-11 | 0.01167 | 0.00540 | 3.08E-02 | Yes |
| 4 | rs7528419 | 1 | 109817192 | *CELSR2* | G | A | 0.224671 | 0.0197401 | 0.00238729 | 1.35E-16 | 0.01790 | 0.00645 | 5.47E-03 | Yes |
| 5 | rs1933064 | 1 | 152301576 | *FLG-AS1* | A | G | 0.46961 | 0.015731 | 0.00203195 | 9.80E-15 | 0.01551 | 0.00539 | 4.04E-03 | Yes |
| 6 | rs76798800 | 1 | 154994978 | *DCST2* | G | T | 0.733745 | 0.0121989 | 0.00225962 | 6.71E-08 | 0.01739 | 0.00617 | 4.83E-03 | Yes |
| 7 | rs6672758 | 1 | 230303512 | *GALNT2* | T | C | 0.800872 | 0.0175857 | 0.00250898 | 2.40E-12 | 0.01563 | 0.00666 | 1.90E-02 | Yes |
| 8 | rs727857 | 2 | 58981967 | *LINC01122* | G | A | 0.388511 | 0.0140184 | 0.00206152 | 1.05E-11 | 0.01091 | 0.00551 | 4.75E-02 | Yes |
| 9 | rs1047891 | 2 | 211540507 | *CPS1* | C | A | 0.684179 | 0.0152142 | 0.00214041 | 1.18E-12 | 0.01266 | 0.00573 | 2.71E-02 | Yes |
| 10 | rs2012736 | 2 | 234622379 | *UGT1A5, UGT1A6, UGT1A7, UGT1A8, UGT1A9, UGT1A10* | C | A | 0.919186 | 0.0483073 | 0.00366555 | 1.16E-39 | 0.03844 | 0.01038 | 2.13E-04 | Yes |
| 11 | rs6782190 | 3 | 85639672 | *CADM2* | G | A | 0.352488 | 0.0172156 | 0.00208415 | 1.45E-16 | 0.02067 | 0.00562 | 2.38E-04 | Yes |
| 12 | rs705117 | 4 | 72608115 | *GC* | C | T | 0.1477 | 0.0334179 | 0.00280601 | 1.06E-32 | 0.02694 | 0.00744 | 2.94E-04 | Yes |
| 13 | rs1352846 | 4 | 72617775 | *GC* | A | G | 0.708567 | 0.193471 | 0.00219074 | 0 | 0.22218 | 0.00590 | 1.40E-310 | Yes |
| 14 | rs78151190 | 6 | 25619007 | *CARMIL1 (LRRC16A)* | A | C | 0.871284 | 0.0168754 | 0.00297406 | 1.39E-08 | 0.01871 | 0.00830 | 2.41E-02 | Yes |
| 15 | rs75741381 | 7 | 100809458 | *VGF* | C | G | 0.852362 | 0.0166065 | 0.00282521 | 4.15E-09 | 0.02145 | 0.00736 | 3.58E-03 | Yes |
| 16 | rs12056768 | 8 | 116988527 | *LINC00536* | T | G | 0.417091 | 0.0234029 | 0.00202418 | 6.44E-31 | 0.01766 | 0.00545 | 1.20E-03 | Yes |
| 17 | rs77532868 | 10 | 88081438 | *GRID1* | T | C | 0.054042 | 0.0265692 | 0.00440069 | 1.57E-09 | 0.02806 | 0.01353 | 3.81E-02 | Yes |
| 18 | rs12794714 | 11 | 14913575 | *CYP2R1* | G | A | 0.578197 | 0.0878964 | 0.00201629 | 0 | 0.07025 | 0.00540 | 1.22E-38 | Yes |
| 19 | rs61891388 | 11 | 66079818 | *RP11-867G23.13* | G | T | 0.455921 | 0.0125532 | 0.00200799 | 4.06E-10 | 0.01143 | 0.00539 | 3.40E-02 | Yes |
| 20 | rs1660839 | 11 | 71094232 | *AP002387.1* | A | G | 0.248849 | 0.0292665 | 0.00230557 | 6.40E-37 | 0.01417 | 0.00624 | 2.30E-02 | Yes |
| 21 | rs12803256 | 11 | 71132868 | *AP002387.1* | G | A | 0.776732 | 0.104243 | 0.00239998 | 0 | 0.08391 | 0.00603 | 4.39E-44 | Yes |
| 22 | rs12798050^4^ | 11 | 71223256 | *S100A11P3* | T | C | 0.830503 | 0.109998 | 0.00264849 | 0 | 0.03480 | 0.00240 | 1.00E-47 | Yes |
| 23 | rs72997623 | 11 | 75488054 | *DGAT2* | A | C | 0.084662 | 0.0276158 | 0.00358139 | 1.25E-14 | 0.02002 | 0.00938 | 3.28E-02 | Yes |
| 24 | rs1149605 | 11 | 76485216 | *RP11-21L23.4* | C | T | 0.170397 | 0.0220166 | 0.00266133 | 1.31E-16 | 0.02098 | 0.00724 | 3.77E-03 | Yes |
| 25 | rs10859995 | 12 | 96375682 | *HAL* | T | C | 0.417366 | 0.0403465 | 0.0020206 | 1.05E-88 | 0.03655 | 0.00541 | 1.36E-11 | Yes |
| 26 | rs8018720 | 14 | 39556185 | *SEC23A* | G | C | 0.176673 | 0.0378247 | 0.00260904 | 1.26E-47 | 0.04085 | 0.00705 | 6.91E-09 | Yes |
| 27 | rs261291 | 15 | 58680178 | *ALDH1A2* | T | C | 0.644772 | 0.0273653 | 0.00208561 | 2.50E-39 | 0.01135 | 0.00564 | 4.41E-02 | Yes |
| 28 | rs77924615 | 16 | 20392332 | *PDILT* | G | A | 0.806515 | 0.0166321 | 0.00255158 | 7.11E-11 | 0.01955 | 0.00671 | 3.55E-03 | Yes |
| 29 | rs212100 | 19 | 48376995 | *SULT2A1* | T | C | 0.164001 | 0.0661522 | 0.00269018 | 1.61E-133 | 0.01939 | 0.00720 | 7.06E-03 | Yes |
| 30 | rs10426 | 19 | 51517798 | *KLK10* | A | G | 0.213433 | 0.0256629 | 0.00243056 | 4.64E-26 | 0.01464 | 0.00654 | 2.51E-02 | Yes |
| 31 | rs6123359 | 20 | 52714706 | *BCAS1* | G | A | 0.102225 | 0.0341831 | 0.00331429 | 6.10E-25 | 0.03736 | 0.00940 | 7.08E-05 | Yes |
| 32 | rs17216707 | 20 | 52732362 | *CYP24A1* | T | C | 0.817316 | 0.0376264 | 0.00263713 | 3.47E-46 | 0.06469 | 0.00664 | 2.02E-22 | Yes |
| 33 | rs2585442 | 20 | 52737123 | *CYP24A1* | G | C | 0.240654 | 0.0356675 | 0.00237687 | 6.70E-51 | 0.03815 | 0.00636 | 1.96E-09 | Yes |
| 34 | rs2762943 | 20 | 52790786 | *CYP24A1* | G | T | 0.923071 | 0.0457231 | 0.00373798 | 2.10E-34 | 0.03253 | 0.01442 | 2.41E-02 | Yes |
| 35 | rs2074735 | 22 | 31535872 | *PLA2G3* | C | G | 0.064096 | 0.0278196 | 0.00407045 | 8.23E-12 | 0.02137 | 0.01055 | 4.27E-02 | Yes |
| 36 | rs11591147 | 1 | 55505647 | *PCSK9* | T | G | 0.018041 | 0.0450903 | 0.00747745 | 1.64E-09 | -0.01378 | 0.02994 | 6.45E-01 | No |
| 37 | rs2131925 | 1 | 63025942 | *DOCK7* | G | T | 0.356375 | 0.0229402 | 0.0020845 | 3.61E-28 | -0.00675 | 0.00571 | 2.37E-01 | No |
| 38 | rs140371183 | 1 | 152098428 | *PUDPP2 (HDHD1P2)* | G | A | 0.032179 | 0.0870147 | 0.00565335 | 1.86E-53 | - | - | - | No |
| 39 | rs12123821 | 1 | 152179152 | *FLG-AS1, RP11-107M16.2* | T | C | 0.047527 | 0.0785529 | 0.00467652 | 2.55E-63 | - | - | - | No |
| 40 | rs61816761 | 1 | 152285861 | *FLG-AS1, FLG* | A | G | 0.015926 | 0.12315 | 0.00804767 | 7.35E-53 | - | - | - | No |
| 41 | rs10908419 | 1 | 154567699 | *ADAR* | G | A | 0.510067 | 0.012342 | 0.00199206 | 5.81E-10 | 0.00562 | 0.00536 | 2.95E-01 | No |
| 42 | rs11264322 | 1 | 155087933 | *Y_RNA* | G | A | 0.570235 | 0.0093848 | 0.0020223 | 3.47E-06 | -0.00399 | 0.00540 | 4.60E-01 | No |
| 43 | rs10908465 | 1 | 155389688 | *ASH1L* | T | C | 0.267332 | 0.0168816 | 0.00224924 | 6.12E-14 | 0.01189 | 0.00608 | 5.04E-02 | No |
| 44 | rs867772 | 1 | 220972343 | *MARC1* | A | G | 0.315487 | 0.0146031 | 0.00215141 | 1.14E-11 | 0.00409 | 0.00577 | 4.79E-01 | No |
| 45 | rs7604788 | 2 | 21190024 | *RP11-116D2.1* | T | C | 0.033433 | 0.0336737 | 0.0055518 | 1.32E-09 | 0.00321 | 0.01348 | 8.12E-01 | No |
| 46 | rs541041 | 2 | 21294975 | *APOB* | G | A | 0.180761 | 0.0154585 | 0.00258635 | 2.27E-09 | 0.00949 | 0.00693 | 1.71E-01 | No |
| 47 | rs1260326 | 2 | 27730940 | *GCKR* | C | T | 0.606565 | 0.0206128 | 0.00203644 | 4.41E-24 | 0.00026 | 0.00545 | 9.62E-01 | No |
| 48 | rs11127186 | 2 | 28881407 | *AC074011.2* | C | T | 0.495795 | 0.0109023 | 0.00203379 | 8.30E-08 | 0.00329 | 0.00537 | 5.40E-01 | No |
| 49 | rs2710651 | 2 | 63166379 | *EHBP1* | G | A | 0.471877 | 0.0114568 | 0.00199623 | 9.51E-09 | 0.00544 | 0.00537 | 3.11E-01 | No |
| 50 | rs3849374 | 2 | 101443397 | *NPAS2, AC092168.2* | G | C | 0.821971 | 0.0161021 | 0.00261564 | 7.46E-10 | 0.00645 | 0.00682 | 3.44E-01 | No |
| 51 | rs7569755 | 2 | 118648261 | *HTR5BP* | A | G | 0.29058 | 0.01425 | 0.00221206 | 1.18E-10 | 0.00450 | 0.00599 | 4.53E-01 | No |
| 52 | rs13060130 | 3 | 84440527 | *AC108696.1* | C | T | 0.860311 | 0.0149893 | 0.00287224 | 1.80E-07 | -0.00734 | 0.00765 | 3.37E-01 | No |
| 53 | rs9861009 | 3 | 141654685 | *TFDP2 (RP11-271K21.11)* | C | T | 0.727515 | 0.0140213 | 0.00225294 | 4.86E-10 | 0.00893 | 0.00600 | 1.37E-01 | No |
| 54 | rs78649910 | 4 | 3482213 | *DOK7* | T | A | 0.893821 | 0.0211949 | 0.00325203 | 7.15E-11 | 0.00008 | 0.00882 | 9.93E-01 | No |
| 55 | rs4364259 | 4 | 15892159 | *RP11-442P12.1* | A | G | 0.202148 | 0.0159119 | 0.00250595 | 2.16E-10 | 0.00658 | 0.00701 | 3.48E-01 | No |
| 56 | rs4616820 | 4 | 57745481 | *REST* | C | T | 0.535046 | 0.012286 | 0.00201755 | 1.13E-09 | 0.00060 | 0.00539 | 9.12E-01 | No |
| 57 | rs35057908 | 4 | 69372082 | *UGT2B29P* | T | A | 0.431309 | 0.0110085 | 0.00202343 | 5.31E-08 | - | - | - | No |
| 58 | rs13104260 | 4 | 70348090 | *UGT2B4* | A | G | 0.256938 | 0.0072531 | 0.00228347 | 0.00149139 | -0.00141 | 0.00624 | 8.22E-01 | No |
| 59 | rs11732896 | 4 | 88287993 | *HSD17B11* | G | A | 0.701209 | 0.0160047 | 0.00217341 | 1.79E-13 | 0.00489 | 0.00592 | 4.09E-01 | No |
| 60 | rs28364331 | 4 | 100201295 | *RP11-696N14.1, ADH1A* | G | A | 0.018086 | 0.068614 | 0.00747169 | 4.19E-20 | 0.02169 | 0.02246 | 3.34E-01 | No |
| 61 | rs1229984 | 4 | 100239319 | *ADH1B* | T | C | 0.024889 | 0.0450574 | 0.00637113 | 1.53E-12 | - | - | - | No |
| 62 | rs10070734 | 5 | 87940026 | *LINC00461* | C | T | 0.709531 | 0.0132137 | 0.00219753 | 1.82E-09 | 0.00608 | 0.00587 | 3.00E-01 | No |
| 63 | rs31612 | 5 | 108996643 | *AC012603.1* | T | C | 0.825562 | 0.014528 | 0.00264902 | 4.15E-08 | -0.00046 | 0.00691 | 9.47E-01 | No |
| 64 | rs72834856 | 6 | 22801858 | *RP1-209A6.1* | T | G | 0.927936 | 0.0249871 | 0.00385103 | 8.67E-11 | 0.00405 | 0.01030 | 6.94E-01 | No |
| 65 | rs28374650 | 6 | 32623367 | *HLA-DQB1* | C | T | 0.756438 | 0.0135623 | 0.00232502 | 5.44E-09 | -0.00718 | 0.00604 | 2.35E-01 | No |
| 66 | rs9476310 | 6 | 57767576 | *RP11-325M4.2* | T | C | 0.511363 | 0.0117571 | 0.00200093 | 4.21E-09 | 0.00077 | 0.00536 | 8.86E-01 | No |
| 67 | rs9490317 | 6 | 121859499 | *RNU4-76P* | C | T | 0.445894 | 0.011051 | 0.00201182 | 3.95E-08 | 0.00863 | 0.00542 | 1.12E-01 | No |
| 68 | rs2248551 | 6 | 131924689 | *MED23* | G | A | 0.834778 | 0.0233623 | 0.0026823 | 3.04E-18 | 0.00901 | 0.00738 | 2.22E-01 | No |
| 69 | rs10085881 | 7 | 21577960 | *DNAH11* | T | C | 0.717815 | 0.0145575 | 0.00223829 | 7.83E-11 | -0.00239 | 0.00601 | 6.92E-01 | No |
| 70 | rs7784802 | 7 | 64015379 | *ZNF680* | T | A | 0.360991 | 0.0138131 | 0.00207209 | 2.62E-11 | 0.00626 | 0.00564 | 2.67E-01 | No |
| 71 | rs6966728 | 7 | 104618318 | *LINC01004* | C | T | 0.537368 | 0.0117532 | 0.00203758 | 8.01E-09 | -0.00077 | 0.00537 | 8.86E-01 | No |
| 72 | rs2346264 | 7 | 133536351 | *EXOC4* | A | C | 0.217315 | 0.0138826 | 0.00243596 | 1.21E-08 | -0.00474 | 0.00649 | 4.65E-01 | No |
| 73 | rs34290760 | 8 | 9185179 | *RP11-115J16.1* | C | G | 0.970888 | 0.0334615 | 0.00593241 | 1.70E-08 | 0.02002 | 0.01633 | 2.20E-01 | No |
| 74 | rs804281 | 8 | 11611865 | *GATA4* | G | A | 0.583605 | 0.0132996 | 0.00202139 | 4.72E-11 | 0.00296 | 0.00544 | 5.86E-01 | No |
| 75 | rs28692966 | 8 | 25892919 | *EBF2* | A | G | 0.252936 | 0.0148311 | 0.00230018 | 1.14E-10 | 0.00378 | 0.00613 | 5.37E-01 | No |
| 76 | rs2725371 | 8 | 30854033 | *PURG* | G | A | 0.697691 | 0.0118392 | 0.00218287 | 5.84E-08 | -0.00675 | 0.00585 | 2.48E-01 | No |
| 77 | rs4738684 | 8 | 59393273 | *CYP7A1* | G | A | 0.665522 | 0.0124102 | 0.00211491 | 4.41E-09 | 0.00462 | 0.00565 | 4.13E-01 | No |
| 78 | rs13284054 | 9 | 107669073 | *ABCA1* | C | T | 0.117727 | 0.0175688 | 0.00313411 | 2.07E-08 | 0.00148 | 0.00838 | 8.60E-01 | No |
| 79 | rs10887718 | 10 | 82042624 | *MAT1A* | C | T | 0.471787 | 0.0111219 | 0.00199828 | 2.61E-08 | -0.00079 | 0.00537 | 8.84E-01 | No |
| 80 | rs3925446 | 10 | 91495322 | *KIF20B* | A | G | 0.199129 | 0.0152166 | 0.00249607 | 1.09E-09 | 0.01144 | 0.00669 | 8.73E-02 | No |
| 81 | rs4418728 | 10 | 94839724 | *CYP26A1* | T | G | 0.451688 | 0.0109611 | 0.00200006 | 4.24E-08 | 0.00366 | 0.00537 | 4.95E-01 | No |
| 82 | rs61883501 | 11 | 13882754 | *RP11-98J9.2, RP11-98J9.3* | A | C | 0.966768 | 0.0017709 | 0.00553993 | 0.749232 | 0.02644 | 0.01571 | 9.23E-02 | No |
| 83 | rs116970203 | 11 | 14876718 | *PDE3B* | G | A | 0.972844 | 0.376873 | 0.00612043 | 0 | - | - | - | No |
| 84 | rs117576073 | 11 | 14912573 | *CYP2R1* | G | T | 0.987265 | 0.147177 | 0.00886081 | 5.91E-62 | - | - | - | No |
| 85 | rs78168201 | 11 | 70971149 | *SHANK2* | T | C | 0.013766 | 0.0893469 | 0.00864963 | 5.18E-25 | - | - | - | No |
| 86 | rs964184 | 11 | 116648917 | *ZPR1 (ZNF259)* | C | G | 0.868376 | 0.0431755 | 0.00294423 | 1.09E-48 | 0.00854 | 0.00774 | 2.70E-01 | No |
| 87 | rs613808 | 11 | 116710968 | *APOA1-AS* | G | A | 0.719993 | 0.0264476 | 0.00223913 | 3.40E-32 | 0.01051 | 0.00586 | 7.28E-02 | No |
| 88 | rs2847500 | 11 | 120114421 | *POU2F3* | G | A | 0.876497 | 0.021925 | 0.00302751 | 4.42E-13 | 0.01350 | 0.00821 | 1.00E-01 | No |
| 89 | rs12317268 | 12 | 21352541 | *SLCO1B1* | A | G | 0.848996 | 0.0208967 | 0.00278474 | 6.19E-14 | 0.01296 | 0.00729 | 7.54E-02 | No |
| 90 | rs11182428 | 12 | 38526387 | *RNA5SP358* | T | C | 0.480005 | 0.0125352 | 0.00199379 | 3.23E-10 | 0.00932 | 0.00537 | 8.26E-02 | No |
| 91 | rs1038165 | 12 | 68665940 | *MDM1* | T | C | 0.583349 | 0.0120567 | 0.002018 | 2.31E-09 | 0.00430 | 0.00540 | 4.26E-01 | No |
| 92 | rs11108368 | 12 | 96386138 | *HAL* | G | A | 0.606145 | 0.0038307 | 0.00209129 | 0.0669916 | - | - | - | No |
| 93 | rs12372115 | 12 | 97982701 | *RMST* | G | T | 0.929281 | 0.0217954 | 0.00387948 | 1.93E-08 | -0.00463 | 0.01036 | 6.55E-01 | No |
| 94 | rs73413596 | 12 | 111582630 | *CUX2* | C | T | 0.073854 | 0.0216996 | 0.00382584 | 1.41E-08 | 0.00924 | 0.01018 | 3.64E-01 | No |
| 95 | rs7149014 | 14 | 29802911 | *RP11-562L8.1* | T | C | 0.370807 | 0.0129475 | 0.00208615 | 5.42E-10 | -0.00086 | 0.00554 | 8.77E-01 | No |
| 96 | rs12881545 | 14 | 101176212 | *DLK1* | C | G | 0.673439 | 0.011822 | 0.00213142 | 2.91E-08 | 0.00086 | 0.00565 | 8.80E-01 | No |
| 97 | rs1800588 | 15 | 58723675 | *ALDH1A2, LIPC* | C | T | 0.784797 | 0.0329215 | 0.00242187 | 4.38E-42 | 0.00705 | 0.00654 | 2.82E-01 | No |
| 98 | rs55829990 | 15 | 63790642 | *USP3* | T | C | 0.655996 | 0.0186013 | 0.00210294 | 9.12E-19 | -0.00083 | 0.00559 | 8.82E-01 | No |
| 99 | rs62007299 | 15 | 77711719 | *PEAK1* | G | A | 0.287463 | 0.0133407 | 0.00219977 | 1.32E-09 | 0.00908 | 0.00594 | 1.26E-01 | No |
| 100 | rs325384 | 15 | 100229761 | *MEF2A* | C | T | 0.715795 | 0.0141728 | 0.00221789 | 1.66E-10 | 0.00868 | 0.00597 | 1.46E-01 | No |
| 101 | rs17231506 | 16 | 56994528 | *CETP* | C | T | 0.676894 | 0.0184236 | 0.00213148 | 5.45E-18 | 0.00320 | 0.00572 | 5.76E-01 | No |
| 102 | rs11076175 | 16 | 57006378 | *CETP* | G | A | 0.178358 | 0.0230493 | 0.00260705 | 9.47E-19 | 0.00824 | 0.00732 | 2.61E-01 | No |
| 103 | rs4327060 | 16 | 72807438 | *RP5-991G20.1* | C | T | 0.945604 | 0.0243589 | 0.00439221 | 2.92E-08 | 0.00682 | 0.01166 | 5.58E-01 | No |
| 104 | rs4575545 | 16 | 79755446 | *RP11-345M22.1, RP11-345M22.2* | G | A | 0.695172 | 0.0155823 | 0.00217312 | 7.47E-13 | 0.00853 | 0.00577 | 1.39E-01 | No |
| 105 | rs11542462 | 16 | 82033810 | *SDR42E1* | G | A | 0.865656 | 0.023334 | 0.00291776 | 1.27E-15 | 0.01467 | 0.00801 | 6.72E-02 | No |
| 106 | rs10454087 | 17 | 40735641 | *RETREG3 (FAM134C)* | C | T | 0.715178 | 0.0135306 | 0.00220667 | 8.70E-10 | 0.00290 | 0.00604 | 6.31E-01 | No |
| 107 | rs2952289 | 17 | 66464414 | *RP11-120M18.2* | T | C | 0.798032 | 0.017715 | 0.00249217 | 1.18E-12 | 0.00359 | 0.00667 | 5.90E-01 | No |
| 108 | rs8091117 | 18 | 28919794 | *DSG1* | C | A | 0.934702 | 0.0263626 | 0.0040284 | 5.98E-11 | -0.00162 | 0.01025 | 8.75E-01 | No |
| 109 | rs4121823 | 18 | 47144223 | *LIPG* | T | A | 0.154667 | 0.0192879 | 0.00277797 | 3.83E-12 | 0.00779 | 0.00754 | 3.02E-01 | No |
| 110 | rs590215 | 18 | 57904088 | *RP11-795H16.2* | C | T | 0.734073 | 0.0129217 | 0.00225753 | 1.04E-08 | 0.00355 | 0.00608 | 5.60E-01 | No |
| 111 | rs2037511 | 18 | 61366207 | *SERPINB11* | A | G | 0.166007 | 0.0181228 | 0.00267963 | 1.35E-11 | 0.01096 | 0.00721 | 1.29E-01 | No |
| 112 | rs142158911 | 19 | 11190534 | *LDLR* | A | G | 0.114608 | 0.0255317 | 0.00314553 | 4.79E-16 | 0.00067 | 0.00914 | 9.41E-01 | No |
| 113 | rs187429064 | 19 | 19380513 | *AC138430.4, TM6SF2* | G | A | 0.011266 | 0.0648324 | 0.00947915 | 7.95E-12 | - | - | - | No |
| 114 | rs3814995 | 19 | 36342212 | *NPHS1* | C | T | 0.688405 | 0.012558 | 0.00214992 | 5.18E-09 | - | - | - | No |
| 115 | rs7412 | 19 | 45412079 | *APOE* | T | C | 0.082073 | 0.0300485 | 0.00363434 | 1.36E-16 | -0.00121 | 0.01025 | 9.06E-01 | No |
| 116 | rs484195^5^ | 19 | 45421877 | *APOC1* | A | G | 0.384386 | 0.0155156 | 0.00209699 | 1.37E-13 | 0.00160 | 0.00280 | 5.62E-01 | No |
| 117 | rs8113404 | 19 | 53065579 | *ZNF808, ZNF701* | T | C | 0.304586 | 0.012173 | 0.00217136 | 2.07E-08 | 0.00672 | 0.00581 | 2.47E-01 | No |
| 118 | rs11606 | 19 | 54658102 | *CNOT3* | G | C | 0.425162 | 0.0120363 | 0.00205119 | 4.41E-09 | 0.00807 | 0.00551 | 1.43E-01 | No |
| 119 | rs2207132 | 20 | 39142516 | *MAFB* | G | A | 0.96711 | 0.0345955 | 0.00557773 | 5.56E-10 | -0.00828 | 0.01540 | 5.91E-01 | No |
| 120 | rs2229742 | 21 | 16339172 | *NRIP1* | G | C | 0.896549 | 0.0251483 | 0.00327069 | 1.48E-14 | 0.01442 | 0.00851 | 9.02E-02 | No |
| 121 | rs6003456 | 22 | 23356100 | *AP000362.1* | T | A | 0.765336 | 0.013277 | 0.00236378 | 1.95E-08 | -0.00146 | 0.00632 | 8.17E-01 | No |
| 122 | rs115621755 | 22 | 50853134 | *PPP6R2* | C | T | 0.67288 | 0.0124309 | 0.00212296 | 4.76E-09 | - | - | - | No |

A1: serum-25(OH)D-increasing allele; A2: alternative allele; A1F: allele frequency for A1; SE: standard error; SNP: single nucleotide polymorphism; CHR: chromosome number; BP: base-pair position, Genome Reference Consortium Human Build 37 (GRCh37); vitaminD-GRS: genetic instrument (using 35 GWAS variants) for serum 25(OH)D concentration used in the primary analysis; ^1^estimates of the 122 variants-25(OH)D association from UK Biobank. **^2^**imputed summary statistics, obtained from Revez, 2020 et al.(7), serum 25(OH)D (nmol/L) has been natural-log transformed; ^3^SNP proxy in the SUNLIGHT consortium: rs1123571, r^2^ = 0.86806 (1000 Genome, EUR); ^4^SNP proxy in the SUNLIGHT consortium: rs2186777, r^2^ = 1 (1000 Genome, EUR). ^5^SNP proxy in the SUNLIGHT consortium: rs439401, r2 = 0.87945 (1000 Genome, EUR). Estimates were not found (-) for SNP or its proxy that were not available in SUNLIGHT consortium.

**Supplementary Table 4:** Functional blocks used in the leave-block-out analyses.

| **Functional block^1^** | **SNP** | **Gene** | **Traits** |
| --- | --- | --- | --- |
| **Blood traits** | rs78151190 | CARMIL1 (LRRC16A) | Various, incl. hemoglobin, platelet count, reticulocyte count |
|  | rs1352846 | GC | White cell count, granulocyte count |
|  | rs1047891 | CPS1 | Various, incl. amino acid levels, platelet count, hemoglobin, metabolite levels |
|  | rs72997623 | DGAT2 | Hemoglobin |
|  | rs7528419 | CELSR2 | Blood protein levels, progranulin levels |
|  | rs6672758 | GALNT2 | Platelet count, red cell distribution |
| **Metabolic** | rs76798800 | DCST2 | Various, incl. fat-free mass, birth weight, height |
|  | rs78151190 | CARMIL1 (LRRC16A) | HbA1c, pulse, blood pressure |
|  | rs77924615 | PDILT | Blood pressure |
|  | rs261291 | ALDH1A2 | Cholesterol, lipid metabolism |
|  | rs727857 | LINC01122 | Fat mass, BMI, impedance, weight |
|  | rs1047891 | CPS1 | Various, incl. weight, impedance, fat- free mass cholesterol |
|  | rs12794714 | CYP2R1 | Hip circumference |
|  | rs72997623 | DGAT2 | HDL cholesterol |
|  | rs7528419 | CELSR2 | Angina pectoris, coronary artery disease, cholesterol, statin use |
|  | rs75741381 | VGF | Impedance |
|  | rs6782190 | CADM2 | Fat free mass, BMI, impedance |
| **Renal** | rs77924615 | PDILT | Glomerular filtration rate |
|  | rs1047891 | CPS1 | Creatinine, chronic kidney disease |
|  | rs17216707 | CYP24A1 | Creatinine, glomerular filtration rate |
| **Unclassified** | rs6782190 | CADM2 | Nervous feelings, alcohol intake, smoking, risk taking, number of children fathered |
|  | rs61891388 | RP11-867G23.13 | Qualifications |
|  | rs12803256 | AP002387.1 | Population differentiation |
|  | rs1047891 | CPS1 | Headache |
|  | rs212100 | SULT2A1 | Cholelithiasis, dihydroepiandrosterone sulphate |
|  | rs7522116 | FOXO6 | Intelligence, qualifications |
|  | rs78151190 | CARMIL1 (LRRC16A) | Disorders of mineral metabolism |
|  | rs261291 | ALDH1A2 | Age-related macular degeneration |
|  | rs12056768 | LINC00536 | Hair or balding pattern: pattern 4 |
|  | rs1933064 | FLG-AS1 | Atopic dermatitis |

^1^Functional blocks identified using trait associations identified through PhenoScanner V2 (16).

**Supplementary Table 5:** Summary of 25(OH)D, Total Brain Volume (TBV), White Matter Hyperintensity volume (WMH), the incidence of dementia and stroke, by baseline characteristics of the UK Biobank cohort

|  | N (%) | 25(OH)D median (IQR) | TBV median (IQR)^2,3^ | WMH median (IQR)^3,4^ | Incident dementia rate (n)^5,6,7^ | Incident stroke rate (n) ^5,6,7^ |
| --- | --- | --- | --- | --- | --- | --- |
| Sex |  |  |  |  |  |  |
| Male | 199,275 (46.6) | 47.0 (32.3, 62.3) | 1483.4 (1435.6, 1530.5) | 9.7 (9.0, 10.4) | 9.0 (1,459) | 14.7 (3,172) |
| Female | 228,415 (53.4) | 46.8 (32.5, 62.6) | 1508.1 (1457.3, 1557.6) | 10.3 (9.6, 10.9) | 5.8 (1,955) | 8.7 (2,167) |
| P^1^ |  | $1.7{\times10}^{-4}$ | ${2.6\times10}^{-187}$ | 0.82 | ${1.3\times10}^{-35}$ | ${6.3\times10}^{-78}$ |
| Age |  |  |  |  |  |  |
| 40-49 years | 102,588 (24.0) | 43.2 (29.2, 59.6) | 1546.2 (1505.8, 1587.0) | 2.0 (1.2, 3.4) | 1.0 (112) | 3.7 (411) |
| 50-59 years | 142,304 (33.3) | 45.6 (31.4, 61.4) | 1497.5 (1454.9, 1540.0) | 3.6 (2.1, 6.5) | 3.3 (513) | 8.1 (1,253) |
| 60-73 years | 182,798 (42.7) | 49.8 (35.3, 64.5) | 1448.8 (1408.0, 1490.5) | 6.5 (3.5, 12.2) | 14.1 (2,789) | 18.7 (3,675) |
| P^1^ |  | ${<1.0\times10}^{-300}$ | ${<1.0\times10}^{-300}$ | ${<1.0\times10}^{-300}$ | ${<1.0\times10}^{-300}$ | ${<1.0\times10}^{-300}$ |
| Ethnic background |  |  |  |  |  |  |
| British, Irish or White | 403,475 (94.3) | 47.9 (20.9, 63.2) | 1496.0 (1446.0, 1544.9) | 3.6 (1.9, 7.2) | 7.4 (3,250) | 11.6 (5,103) |
| Indian, Pakistani, Bangladeshi or Asian | 7,709 (1.8) | 23.6 (16.3, 35.1) | 1507.4 (1450.2, 1567.8) | 3.1 (2.0, 4.6) | 3.4 (47) | 8.3 (71) |
| African, Caribbean or Black | 6,793 (1.6) | 30.4 (21.6, 42.2) | 1521.7 (1481.1, 1572.0) | 3.4 (2.1, 6.1) | 5.7 (58) | 8.8 (73) |
| Chinese | 1,362 (0.3) | 32.3 (24.1, 43.8) | 1502.0 (1458.2, 1543.9) | 3.1 (2.0, 4.6) | 8.2 (5) | 10.3 (12) |
| Mixed or other ethnic groups | 6,366 (1.5) | 33.7 (23.0, 47.9) | 1507.2 (1454.1, 1554.8) | 3.0 (1.7, 5.6) | 4.3 (30) | 8.5 (58) |
| Missing | 1,985 (0.5) | 37.6 (24.5, 53.8) | 1490.2 (1439.0, 1532.9) | 4.0 (2.4, 7.8) | 11.3 (24) | 10.5 (22) |
| P^1^ |  | ${<1.0\times10}^{-300}$ | $5.7{\times10}^{-3}$ | 0.32 | ${<1.0\times10}^{-300}$ | 0.062 |
| Skin colour |  |  |  |  |  |  |
| Very fair | 32,549 (7.6) | 41.9 (28.8, 57.2) | 1504.6 (1453.6, 1553.0) | 3.4 (1.8, 7.0) | 8.9 (312) | 12.2 (432) |
| Fair | 287,834 (67.1) | 47.5 (33.3, 62.8) | 1494.7 (1444.5, 1544.1) | 3.7 (2.0, 7.3) | 7.5 (2,353) | 11.9 (3,713) |
| Light olive | 78,357 (18.3) | 50.1 (35.1, 65.4) | 1498.1 (1448.3, 1545.8) | 3.4 (1.8, 6.8) | 5.5 (476) | 9.9 (837) |
| Dark olive/brown/black | 23,358 (5.4) | 34.9 (22.8, 52.4) | 1505.5 (1454.8, 1552.1) | 3.5 (1.9, 6.3) | 7.5 (186) | 10.7 (263) |
| Missing | 6,592 (1.5) | 41.7 (27.5, 58.3) | 1482.5 (1431.7, 1522.1) | 4.3 (2.3, 7.9) | 12.0 (87) | 13.1 (94) |
| P^1^ |  | ${<1.0\times10}^{-300}$ | ${3.6\times10}^{-3}$ | 0.13 | $3.2{\times10}^{-10}$ | $1.3{\times10}^{-3}$ |
| BMI |  |  |  |  |  |  |
| Underweight <18.5 kg/m^2^ | 2,196 (0.5) | 46.3 (29.6, 65.9) | 1510.8 (1453.3, 1563.4) | 3.0 (1.5, 6.3) | 6.4 (23) | 13.2 (31) |
| Normal [18.5, 25) kg/m^2^ | 140,027 (32.7) | 50.6 (35.0, 66.4) | 1504.5 (1452.5, 1553.4) | 3.1 (1.7, 6.2) | 6.0 (986) | 9.4 (1,425) |
| Overweight (25,30) kg/m^2^ | 180,706 (42.3) | 47.8 (33.5, 62.8) | 1491.5 (1442.1, 1540.6) | 3.8 (2.0, 7.7) | 6.8 (1,405) | 11.5 (2,254) |
| Obese ≥30kg/ m^2^ | 103,161 (24.1) | 41.0 (28.4, 55.5) | 1491.4 (1442.8, 1538.3) | 4.3 (2.3, 8.5) | 8.5 (954) | 14.2 (1,1589) |
| Missing | 1,600 (0.4) | 35.5 (22.6, 51.7) | 1482.7 (1430.3, 1551.1) | 5.3 (2.5, 16.8) | 27.0 (46) | 24.0 (40) |
| P^1^ |  | ${<1.0\times10}^{-300}$ | ${1.6\times10}^{-18}$ | ${1.9\times10}^{-40}$ | $9.9{\times10}^{-7}$ | ${2.5\times10}^{-15}$ |
| Education |  |  |  |  |  |  |
| None | 71,245 (16.7) | 47.8 (32.8, 63.6) | 1474.2 (1430.0, 1519.7) | 5.4 (3.0, 10.3) | 15.2 (1,187) | 18.5 (1,436) |
| Intermediate (NVQ/CSE/A-levels) | 149,049 (34.8) | 47.8 (33.0, 63.4) | 1502.2 (1451.3, 1551.1) | 3.6 (2.0, 7.0) | 6.2 (1,008) | 10.4 (1,750) |
| High (degree/professional) | 202,414 (47.3) | 46.0 (32.0, 61.3) | 1495.5 (1445.6, 1544.4) | 3.5 (1.8, 7.0) | 5.1 (1,127) | 9.5 (2,072) |
| missing | 4,982 (1.2) | 43.0 (28.2, 59.6) | 1490.1 (1431.9, 1529.3) | 5.6 (2.8, 9.8) | 17.1 (92) | 12.3 (81) |
| P^1^ |  | $3.9{\times10}^{-94}$ | ${1.2\times10}^{-4}$ | ${2.0\times10}^{-4}$ | $2.3{\times10}^{-55}$ | ${1.6\times10}^{-21}$ |
| Townsend deprivation index |  |  |  |  |  |  |
| Less deprived | 215,258 (50.3) | 49.8 (35.4, 64.6) | 1494.8 (1444.5, 1543.1) | 3.6 (2.0, 7.4) | 6.5 (1,528) | 10.5 (2,476) |
| Highly deprived | 212,912 (49.6) | 43.8 (29.7, 59.9) | 1498.4 (1448.3, 1548.5) | 3.5 (1.9, 7.0) | 8.2 (1,883) | 12.5 (2,853) |
| Missing | 520 (0.1) | 45.2 (31.4, 61.1) | 1537.9 (1496.4, 1575.4) | 2.7 (1.8, 3.8) | 5.4 (3) | 18.2 (10) |
| P^1^ |  | ${<1.0\times10}^{-300}$ | 0.90 | 0.80 | ${6.0\times10}^{-21}$ | $3.3{\times10}^{-20}$ |
| Employment status |  |  |  |  |  |  |
| Paid employment or self-employed | 248,614 (58.1) | 45.1 (31.3, 60.7) | 1510.1 (1462.0, 1557.2) | 3.0 (1.7, 5.8) | 2.5 (690) | 7.4 (2,005) |
| Retired | 139,710 (32.7) | 51.3 (36.5, 65.9) | 1453.7 (1411.0, 1498.0) | 6.1 (3.3, 11.8) | 15.3 (2,309) | 18.6 (2,795) |
| Unemployed | 37,290 (8.7) | 41.9 (27.4, 59.0) | 1508.1 (1459.0, 1552.3) | 3.2 (1.8, 6.2) | 9.7 (394) | 12.8 (518) |
| Missing | 2,076 (0.5) | 40.4 (26.0, 57.7) | 1507.4 (1466.9, 1550.9) | 3.1 (1.7, 6.8) | 9.4 (21) | 9.4 (21) |
| P^1^ |  | ${<1.0\times10}^{-300}$ | ${2.5\times10}^{-31}$ | ${3.0\times10}^{-21}$ | ${1.0\times10}^{-135}$ | ${1.3\times10}^{-33}$ |
| Type of physical activity |  |  |  |  |  |  |
| None | 26,736 (6.2) | 33.6 (22.7, 49.3) | 1504.3 (1451.8, 1553.0) | 13.9 (2.0, 7.2) | 14.0 (408) | 17.8 (516) |
| Light/moderate | 354,236 (82.8) | 47.1 (32.9, 62.4) | 1493.6 (1443.5, 1543.3) | 7.2 (2.0, 7.5) | 7.3 (2,800) | 11.6 (4,459) |
| Strenuous sport | 44,464 (10.4) | 53.0 (37.9, 68.2) | 1508.4 (1460.8, 1555.4) | 2.5 (1.6, 5.5) | 2.5 (123) | 6.4 (313) |
| Missing | 2,254 (0.5) | 34.0 (31.6, 50.4) | 1468.5 (1430.3, 1511.0) | 34.8 (1.6, 7.6) | 34.9 (83) | 21.3 (51) |
| P^1^ |  | ${<1.0\times10}^{-300}$ | ${1.1\times10}^{-5}$ | ${2.4\times10}^{-6}$ | ${6.2\times10}^{-56}$ | ${4.1\times10}^{-34}$ |
| Time spent outdoors in winter |  |  |  |  |  |  |
| None | - | - | - | - | - | - |
| <2 hours | 309,041 (72.3) | 46.1 (31.9, 61.7) | 1498.5 (1448.0, 1547.5) | 5.9 (1.8, 6.9) | 6.2 (2,086) | 10.5 (3,526) |
| 3-5 hours | 72,399 (16.9) | 50.6 (35.6, 65.7) | 1482.5 (1436.3, 1532.5) | 9.7 (2.3, 8.6) | 10.4 (820) | 14.8 (1,159) |
| >6 hours | 21,264 (4.9) | 49.7 (34.6, 65.5) | 1494.3 (1450.2, 1548.3) | 8.1 (2.1, 7.8) | 9.3 (215) | 14.2 (325) |
| Missing | 25,046 (5.8) | 42.4 (28.6, 58.2) | 1496.8 (1444.9, 1546.6) | 9.5 (2.1, 7.5) | 10.7 (293) | 11.9 (329) |
| P^1^ |  | ${<1.0\times10}^{-300}$ | 0.52 | ${1.3\times10}^{-5}$ | ${1.6\times10}^{-8}$ | ${3.1\times10}^{-3}$ |
| Time spent outdoors in summer |  |  |  |  |  |  |
| None | 871 (0.2) | 31.8 (21.6, 46.8) | 1507.0 (1445.0, 1552.4) | 21.6 (1.6, 4.2) | 21.7 (20) | 14.1 (13) |
| <2 hours | 142,051 (33.2) | 43.3 (29.9, 58.8) | 1503.8 (1454.0, 1551.5) | 5.1 (1.7, 6.1) | 5.2 (802) | 9.1 (1,1392) |
| 3-5 hours | 177,648 (41.5) | 48.5 (33.9, 63.6) | 1492.3 (1441.0, 1542.3) | 7.3 (2.0, 7.8) | 7.3 (1,419) | 11.7 (2,244) |
| >6 hours | 82,238 (19.2) | 51.4 (36.3, 66.8) | 1483.2 (1436.8, 1532.8) | 9.9 (2.2, 8.7) | 10.0 (891) | 15.3 (1,1359) |
| Missing | 24,882 (5.8) | 41 (27.6, 57.0) | 1497.9 (1445.1, 1548.3) | 10.3 (2.0, 7.3) | 10.3 (282) | 12.1 (331) |
| P^1^ |  | $1.3{\times10}^{-227}$ | ${6.2\times10}^{-3}$ | ${1.0\times10}^{-3}$ | ${2.4\times10}^{-9}$ | $2.5{\times10}^{-4}$ |
| Frequency of sun protection use |  |  |  |  |  |  |
| Never goes out in the sunshine | 2,533 (0.6) | 30.7 (20.8, 44.2) | 1500.5 (1451.5, 1547.7) | 22.0 (2.0, 9.3) | 7.5 (60) | 17.3 (47) |
| Never/rarely | 42,940 (10.0) | 38.7 (25.6, 55.3) | 1481.3 (1430.8, 1533.0) | 12.0 (2.2, 8.2) | 6.1 (566) | 16.6 (774) |
| Sometimes | 142,328 (33.3) | 46.7 (32.5, 62.0) | 1490.8 (1442.0, 1539.4) | 7.5 (1.9, 7.3) | 7.0 (1,175) | 12.4 (1,919) |
| Most of the time | 150,926 (35.3) | 48.7 (34.4, 63.7) | 1500.9 (1450.0, 1549.0) | 5.6 (1.8, 6.8) | 9.6 (926) | 9.6 (1,581) |
| Always | 87,612 (20.5) | 48.5 (33.7, 64.1) | 1501.6 (1451.9, 1552.5) | 6.9 (2.0, 7.1) | 6.9 (664) | 10.5 (999) |
| Missing | 1,351 (0.3) | 28.3 (18.7, 43.2) | 1490.8 (1451.3, 1564.4) | 16.6 (1.6, 5.7) | 18.2 (23) | 13.5 (19) |
| P^1^ |  | ${<1.0\times10}^{-300}$ | $6.0{\times10}^{-12}$ | ${9.9\times10}^{-6}$ | ${4.4\times10}^{-35}$ | ${3.5\times10}^{-16}$ |
| Frequency of oily fish consumption |  |  |  |  |  |  |
| Never | 46,286 (10.8) | 41.7 (27.2, 58.6) | 1514.0 (1462.9, 1563.0) | 7.4 (1.7, 6.2) | 7.5 (376) | 12.1 (602) |
| Less than once a week | 141,354 (33.1) | 45.3 (30.9, 61.1) | 1501.6 (1451.0, 1551.0) | 6.1 (1.8, 6.7) | 6.1 (945) | 10.9 (1,683) |
| Once a week | 160,472 (37.5) | 48.1 (33.8, 63.3) | 1492.7 (1442.9, 1541.0) | 7.0 (2.0, 7.3) | 7.0 (1,230) | 11.0 (1,925) |
| Greater than once a week | 76,588 (17.9) | 50.3 (36.0, 64.9) | 1483.9 (1435.5, 1533.3) | 9.6 (2.1, 8.3) | 9.6 (805) | 13.2 (1,093) |
| Missing | 2,990 (0.7) | 37.3 (23.9, 55.4) | 1499.9 (1435.3, 1564.0) | 18.1 (1.7, 5.3) | 18.2 (58) | 11.2 (36) |
| P^1^ |  | ${<1.0\times10}^{-300}$ | ${9.2\times10}^{-11}$ | 0.028 | ${1.9\times10}^{-10}$ | ${2.5\times10}^{-6}$ |
| Frequency of non-oily fish consumption |  |  |  |  |  |  |
| Never | 19,609 (4.6) | 39.7 (25.1, 57.0) | 1512.0 (1458.9, 1560.8) | 9.4 (1.7, 5.8) | 9.5 (201) | 11.0 (233) |
| Less than once a week | 123,607 (28.9) | 45.5 (31.0, 61.4) | 1497.6 (1447.6, 1547.9) | 5.9 (1.9, 6.9) | 6.0 (796) | 11.2 (1,506) |
| Once a week | 211,814 (49.5) | 47.9 (33.6, 63.1) | 1494.1 (1443.9, 1543.3) | 7.2 (1.9, 7.4) | 7.3 (1,681) | 11.4 (2,634) |
| Greater than once a week | 69,924 (16.4) | 48.3 (34.0, 63.6) | 1496.6 (1447.5, 1541.8) | 8.9 (1.9, 7.1) | 8.9 (681) | 12.1 (916) |
| Missing | 2,736 (0.6) | 35.9 (34.0, 53.0) | 1495.6 (1446.6, 1545.5) | 18.8 (1.8, 5.8) | 18.8 (55) | 17.1 (50) |
| P^1^ |  | ${<1.0\times10}^{-300}$ | 0.12 | 0.37 | ${4.8\times10}^{-18}$ | $9.6{\times10}^{-3}$ |
| Diet restrictions |  |  |  |  |  |  |
| No eggs of dairy containing food | 18,144 (4.2) | 44.4 (29.5, 61.1) | 1493.0 (1444.5, 1541.9) | 14.8 (2.1, 8.2) | 15.0 (294) | 13.9 (276) |
| No wheat containing food | 7,461 (1.7) | 47.8 (32.4, 64.6) | 1495.3 (1447.2, 1549.9) | 14.0 (2.0, 6.4) | 14.0 (116) | 17.8 (144) |
| No sugar, sugar containing food or drinks | 70,976 (16.6) | 49.2 (34.4, 64.6) | 1480.0 (1433.5, 1527.9) | 10.1 (2.3, 8.8) | 10.2 (787) | 15.2 (1,172) |
| Eat all of the above | 329,247 (77.0) | 46.5 (32.2, 62.0) | 1499.3 (1448.7, 1548.2) | 6.0 (1.8, 6.8) | 6.1 (2,180) | 10.4 (3,714) |
| Missing | 1,862 (0.4) | 37.3 (23.9, 55.6) | 1478.1 (1435.2, 1524.0) | 18.6 (2.4, 8.2) | 18.7 (37) | 12.1 (33) |
| P^1^ |  | $7.8{\times10}^{-184}$ | ${6.5\times10}^{-3}$ | ${3.0\times10}^{-7}$ | ${2.3\times10}^{-39}$ | ${9.2\times10}^{-13}$ |
| Depression |  |  |  |  |  |  |
| yes | 44,394 (10.4) | 44.0 (29.8, 60.2) | 1495.5 (1445.4, 1544.3) | 3.5 (1.9, 7.0) | 18.4 (887) | 18.4 (887) |
| no | 383,296 (89.6) | 47.2 (32.7, 62.7) | 1503.0 (1453.1, 1553.0) | 3.6 (1.9, 7.2) | 6.0 (2,527) | 6.0 (2,527) |
| P^1^ |  | $7.3{\times10}^{-160}$ | 0.0079 | $7.0{\times10}^{-3}$ | $8.5{\times10}^{-185}$ | $4.5{\times10}^{-56}$ |
| Diet quality |  |  |  |  |  |  |
| 0 | 2,195 (0.5) | 39.4 (26.5, 56.5) | 1482.5 (1434.0, 1534.1) | 4.3 (2.1, 8.2) | 9.7 (23) | 13.2 (31) |
| 1 | 55,426 (13.0) | 43.7 (29.7, 59.7) | 1499.8 (1450.0, 1549.5) | 3.4 (1.8, 6.6) | 7.0 (424) | 12.3 (736) |
| 2 | 151,158 (35.3) | 45.8 (31.4, 61.3) | 1498.4 (1449.1, 1547.9) | 3.5 (1.8, 6.9) | 7.6 (1,080) | 12.1 (1,908) |
| 3 | 142,609 (33.3) | 48.2 (33.9, 63.4) | 1494.8 (1444.4, 1539.2) | 3.6 (2.0, 7.2) | 7.1 (1,098) | 11.0 (1,702) |
| 4 | 60.604 (14.1) | 51.1 (36.4, 66.0) | 1491.1 (1441.2, 1537.2) | 4.0 (2.2, 8.4) | 7.5 (493) | 10.3 (670) |
| Missing | 15,698 (3.7) | 40.3 (26.6, 57.2) | 1496.1 (1443.3, 1544.9) | 3.8 (2.1, 7.1) | 17.4 (296) | 17.2 (292) |
| P^1^ |  | ${<1.0\times10}^{-300}$ | 0.049 | 0.023 | 0.32 | $1.9{\times10}^{-4}$ |
| Any vitamin or mineral supplements |  |  |  |  |  |  |
| Yes | 216,665 (66.3) | 52.0 (37.8, 66.3) | 1489.6 (1440.9, 1539.3) | 3.8 (2.1, 8.9) | 6.7 (1,846) | 11.2 (2,546) |
| No | 209,602 (49.0) | 41.3 (28.4, 57.2) | 1502.8 (1452.3, 1551.2) | 3.3 (1.8, 6.7) | 7.8 (1,535) | 11.8 (2,767) |
| Missing | 1,423 (0.3) | 33.3 (21.4, 49.2) | 1469.7 (1434.4, 1556.9) | 5.2 (2.6, 8.9) | 21.9 (33) | 17.3 (26) |
| P^1^ |  | ${<1.0\times10}^{-300}$ | $3.5{\times10}^{-7}$ | 0.39 | 0.62 | 0.11 |
| Longstanding illness/infirmity/disability |  |  |  |  |  |  |
| Yes | 133,428 (31.2) | 45.0 (30.6, 61.0) | 1485.5 (1437.6, 1535.8) | 4.3 (2.2, 8.8) | 13.3 (1,930) | 17.4 (2,512) |
| No | 238,384 (66.3) | 47.9 (33.4, 63.2) | 1499.4 (1449.2, 1548.2) | 3.4 (1.9, 6.7) | 4.3 (1,328) | 8.7 (2,660) |
| Missing | 10,878 (2.5) | 42.7 (28.8, 58.5) | 1494.3 (1442.9, 1547.5) | 3.7 (2.0, 7.6) | 13.3 (156) | 14.2 (167) |
| P^1^ |  | ${<1.0\times10}^{-300}$ | $2.5{\times10}^{-12}$ | $4.0{\times10}^{-30}$ | $1.1{\times10}^{-150}$ | $1.2{\times10}^{-78}$ |

^1^ P-values for the pattern after adjustment for sex, age, assessment centre and weighted for 1-kinship co-efficient to account for relatedness
^2^ Information shown for imaging sub-sample for TBV only
^3^ Using linear regression
^4^ Information shown for imaging sub-sample for WMH only
^5^ Information shown for dementia and stroke outcomes sub-sample only
^6^ Rate shown for every 10,000 person-years
^7^ Using cox proportional model

Interquartile range (IQR), Total brian volume (TBV), White matter hyperintensity (WMH)

**Supplementary Table 6:** The association between 25(OH)D (per 10 nmol/L) with neuroimaging outcomes and the risk of dementia and stroke with progressive covariate adjustment for males and females.

| Outcome^1^ | Model | Males | | Females | |
| --- | --- | --- | --- | --- | --- |
|  |  | Beta (LCI, UCI) | P | Beta (LCI, UCI) | P |
| TBV (N_males_=14,851; N_females_=16,186) | Basic | 1064.4(532.6, 1596.3) | 1.2 x 10^-6^ | 161.3(-320.8, 643.4) | 0.51 |
|  | Socioeconomic | 1292.9(753.9, 1831.9) | 2.6 x 10^-6^ | 244.3(-242.8, 731.4) | 0.33 |
|  | Lifestyle | 1009(452.3, 1565.7) | 3.8 x 10^-4^ | 329.5(-181.6, 840.7) | 0.21 |
|  | Sun behaviours | 809.9(248.9, 1370.9) | 4.7 x 10^-3^ | 347.4(-167.5, 862.3) | 0.19 |
|  | Illness | 795.3(235.7, 1354.8) | 5.3 x 10^-3^ | 332.5(-182.5, 847.5) | 0.21 |
| GMV (N_males_=14,851; N_females_=16,186) | Basic | 714.9(390.8, 1039) | 1.5 x 10^-5^ | 228.2(-67.4, 523.8) | 0.13 |
|  | Socioeconomic | 869.8(542.1, 1197.6) | 2.0 x 10^-7^ | 297.4(-0.8, 595.6) | 0.05 |
|  | Lifestyle | 604.2(268.1, 940.4) | 4.3 x 10^-4^ | 258.9(-53.4, 571.3) | 0.10 |
|  | Sun behaviours | 542.1(202.6, 881.6) | 1.8 x 10^-3^ | 278.3(-36.5, 593.1) | 0.08 |
|  | Illness | 524.9(186.3, 863.5) | 2.4 x 10^-3^ | 264.3(-50.3, 578.9) | 0.10 |
| WMV (N_males_=14,851; N_females_=16,186) | Basic | 349.5(17.7, 681.3) | 0.04 | -66.9(-365.4, 231.7) | 0.32 |
|  | Socioeconomic | 423.1(87.1, 759.1) | 0.01 | -53.1(-355, 248.8) | 0.66 |
|  | Lifestyle | 404.8(54.7, 754.9) | 0.02 | 70.6(-246.8, 388.1) | 0.73 |
|  | Sun behaviours | 267.8(-84.8, 620.4) | 0.14 | 69.1(-251, 389.2) | 0.66 |
|  | Illness | 270.4(-82, 622.8) | 0.13 | 68.2(-251.8, 388.3) | 0.67 |
| HV (N_males_=14,842; N_females_=16,183) | Basic | 2.7(-6.1, 11.4) | 0.55 | 3.8(-3.7, 11.3) | 0.68 |
|  | Socioeconomic | 5.3(-3.6, 14.2) | 0.24 | 4.8(-2.8, 12.4) | 0.21 |
|  | Lifestyle | 3.1(-6.1, 12.4) | 0.51 | 3.6(-4.4, 11.5) | 0.38 |
|  | Sun behaviours | 0.3(-9, 9.7) | 0.95 | 3.1(-4.9, 11.2) | 0.44 |
|  | Illness | 0.2(-9.1, 9.6) | 0.96 | 2.9(-5.1, 10.9) | 0.48 |
| logWMH^b^ (N_males_=14,311; N_females_=15,678) | Basic | -13.2(-21, -5.3) | 1.0 x 10^-3^ | -3.2(-9.9, 3.6) | 0.36 |
|  | Socioeconomic | -18.3(-26.3, -10.3) | 6.7 x 10^-6^ | -6.2(-13.1, 0.6) | 0.07 |
|  | Lifestyle | -9.9(-18.1, -1.7) | 0.02 | -1.6(-8.7, 5.6) | 0.67 |
|  | Sun behaviours | -11.2(-19.5, -2.9) | 7.9 x 10^-3^ | -2.5(-9.7, 4.6) | 0.49 |
|  | Illness | -11.0(-19.2, -2.7) | 9.1 x 10^-3^ | -2.1(-9.2, 5) | 0.56 |

^1^ Total brain volume (TBV), Grey matter volume (GMV), White matter volume (WMV), White matter hyperintensity volume (WMH), Hippocampal volume (HV). Lower confidence interval (LCI), Upper confidence interval (UCI). Estimates for brain volumes from linear regression, risks of dementia and stroke assessed using Cox proportional hazards models. Adjustments were as follows: **Basic covariates (model 1)**: age, assessment centre, ethnicity, month; **Socioeconomic (model 2)**: model 1+ education, Townsend deprivation index, employment status; **Lifestyle factors (model 3):** model 2+ BMI (categorical), type of physical activity, diet quality, any use of dietary supplements ; **Sun behaviours (model 4):** model 3+ time spent outdoors in summer, time spent outdoors in winter, sun protection; **Illnesses (All covariates):** model 4+ long-standing illnesses/disability/infirmity +depression

^2^ WMH log transformed in analyses due to skewness.

**Supplementary Table 7:** Vitamin D genetic risk score (Vitamin D-GS) association with potential confounders in the UK biobank

|  |  | **Vitamin D-GRS** |
| --- | --- | --- |
|  | N (%) | Mean (SD) |
| **Sex** |  |  |
| Male | 138,845 (47.1) | 38.43 (5.88) |
| Female | 155,669 (52.9) | 38.43 (5.87) |
| P^1^ |  | 0.81 |
| **Age** |  |  |
| 40-49 years | 66,265 (22.5) | 38.41 (5.86) |
| 50-59 years | 97,171 (33.0) | 38.43 (5.87) |
| 60-73 years | 131,078 (44.5) | 38.43 (5.88) |
| P^1^ |  | 0.51 |
| **BMI** |  |  |
| <18.5 kg/m^2^ | 1,454 (0.5) | 38.38 (5.89) |
| [18.5, 25) kg/m^2^ | 96,652 (32.9) | 38.42 (5.87) |
| [25, 30) kg/m^2^ | 125,179 (42.6) | 38.42 (5.88) |
| ≥30 kg/m^2^ | 70,344 (24.0) | 38.45 (5.87) |
| P^1^ |  | 0.89 |
| **Smoking** |  |  |
| Non-smokers | 161,158 (54.9) | 38.42 (5.86) |
| Ex-smokers | 103,151 (35.1) | 38.44 (5.89) |
| Current smokers | 29,211 (10.0) | 38.39 (5.88) |
| P^1^ |  | 0.20 |
| **Alcohol intake** |  |  |
| Non-drinkers | 18,752 (6.4) | 38.52 (5.83) |
| Special occasions | 30,540 (10.4) | 38.37 (5.90) |
| 1-3 times/month | 32,610 (11.1) | 38.39 (5.87) |
| 1 or 2 times/week | 77,904 (26.4) | 38.46 (5.87) |
| 3 or 4 times/week | 71,583 (24.3) | 38.45 (5.87) |
| Daily or almost daily | 62,927 (21.4) | 38.40 (5.88) |
| P^1^ |  | 0.09 |
| **Physical activity** |  |  |
| None | 16,328 (5.6) | 38.47 (5.88) |
| Light/moderate | 246,043 (83.8) | 38.43 (5.87) |
| Vigorous | 31,163 (10.6) | 38.41 (5.89) |
| P^1^ |  | 0.99 |
| **Supplement use** |  |  |
| No | 202,287 (68.9) | 38.44 (5.87) |
| Yes | 91,129 (31.1) | 38.41 (5.88) |
| P^1^ |  | 0.22 |
| **Education** |  |  |
| None | 49,217 (16.9) | 38.45 (5.86) |
| NVQ/CSE/A-levels | 104,591 (35.9) | 38.44 (5.88) |
| Degree/professional | 138,275 (47.3) | 38.41 (5.88) |
| P^1^ |  | 0.63 |
| **Employment** |  |  |
| Unemployed | 22,916 (7.8) | 38.46 (5.83) |
| Retired | 101,486 (34.6) | 38.43 (5.89) |
| Paid employment/self-employed | 169,287 (57.6) | 38.42 (5.87) |
| P^1^ |  | 0.55 |
| **TDI (using median)** |  |  |
| Less deprived (Q1) | 157,688 (53.6) | 38.43 (5.87) |
| Highly deprived (Q2) | 136,477 (46.4) | 38.42 (5.87) |
| P^1^ |  | 0.52 |

NVQ, National Vocational Qualification; CSE, Certificate of Secondary Education; A-levels, Advanced levels; SD, standard deviation; TDI, Townsend deprivation index^1^ P-values have been adjusted for age, sex, genotyping array, birth location, and assessment center

**Supplementary Table 8:** The association between genetically determined 25(OH)D and neuroimaging outcomes and risk of dementia and stroke, using Two Sample MR and non-linear MR analyses.

| Outcome | Sample size | | IVW | | MR-Egger^1^ | | MR-PRESSO^2^ | | Weighted median | | Weighted mode | | P_non-linear_ |
| --- | --- | --- | --- | --- | --- | --- | --- | --- | --- | --- | --- | --- | --- |
|  |  |  | Beta (95% CI) | P | Beta (95% CI) | P | Beta (95% CI) | P | Beta (95% CI) | P | Beta (95% CI) | P |  |
| TBV | 23901 | | 0.96 (-4.21, 6.12) | 0.71 | 2.13 (-4.56, 8.76) | 0.54 | 0.95 (-4.20,6.1) | 0.71 | 2.75 (-2.23,7.73) | 0.27 | 2.53 (-1.86, 6.93) | 0.26 | 0.57 |
| GMV | 23901 | | 0.64 (-2.58, 3.85) | 0.69 | 2.01 (-2.09, 6.11) | 0.34 | 0.63 (-2.58,3.8) | 0.70 | 2.36 (-0.45,5.18) | 0.10 | 1.77 (-0.81, 4.36) | 0.18 | 0.25 |
| WMV | 23901 | | 0.32 (-2.60, 3.24) | 0.83 | 0.09 (-3.68, 3.87) | 0.96 | 0.32 (-2.59,3.23) | 0.83 | 0.51 (-2.61,3.64) | 0.74 | 0.65 (-2.41, 3.72) | 0.67 | 0.19 |
| logWMH | 23083 | | -0.03 (-0.11, 0.04) | 0.39 | 0.02 (-0.07, 0.11) | 0.67 | -0.03 (-0.12,0.04) | 0.40 | 0.05 (-0.02,0.12) | 0.16 | 0.01 (-0.06, 0.09) | 0.69 | 0.51 |
| HV | 23891 | | 0.03 (-0.07, 0.14) | 0.48 | 0.01 (-0.12, 0.13) | 0.94 | 0.03 (-0.07,0.14) | 0.49 | 0.06 (-0.02,0.14) | 0.14 | 0.02 (-0.05, 0.09) | 0.57 | 1.00 |
|  | Cases | Controls | HR (95% CI) | P | HR (95% CI) | P | HR (95% CI) | P | HR (95% CI) | P | HR (95% CI) | P |  |
| Dementia | 2339 | 292175 | 0.87 (0.70, 1.08) | 0.21 | 0.97 (0.73,1.28) | 0.84 | 0.87 (0.71,1.06) | 0.17 | 0.83 (0.63,1.08) | 0.18 | 0.89 (0.70, 1.14) | 0.38 | 5.9 x 10^-03^ |
| Stroke | 3760 | 290741 | 1.07 (0.87, 1.32) | 0.47 | 1.02 (0.78,1.30) | 0.84 | 1.07 (0.87,1.32) | 0.47 | 1.08 (0.87,1.33) | 0.47 | 1.08 (0.88, 1.32) | 0.44 | 0.57 |

^1^MR-Egger P-intercept (for all) > 0.90 suggesting no pleiotropic variants.

^2^MR-PRESSO outlier test did not detect potential pleiotropic outlier variants

Estimates are per log-transformed 25(OH)D [We used variant-25(OH)D association estimates for the two-sample MR analysis from 25(OH)D GWAS that apply log transformation for 25(OH)D data].

P_non-linear:_ is fractional polynomial non-linearity P-value from non-linear MR analysis excluding outlying strata, see Supplementary Figure 2).

Total brain volume (TBV), Grey matter volume (GMV), White matter volume (WMV), White matter hyperintensity volume (WMH), Hippocampal volume (HV). Hazard Ratio (HR), Lower confidence interval (LCI), Upper confidence interval (UCI).

**Supplementary Table 9:** Leave-block-out analysis for non-linear MR analysis of serum 25(OH)D on dementia risk.

| **Excluded functional block** | **SNPs in the functional block** | **P_non-linear_^1^** |
| --- | --- | --- |
| Blood traits | rs1047891, rs1352846, rs6672758, rs72997623, rs7528419 and rs78151190 | 1.00 (0.04)^2^ |
| Metabolic | rs76798800, rs78151190, rs77924615, rs261291, rs727857, rs1047891, rs12794714, rs72997623, rs7528419, rs75741381 and rs6782190 | 0.05 |
| Renal | rs1047891, rs17216707 and rs77924615 | 0.009 |
| Renal or Metabolic | rs76798800, rs78151190, rs77924615, rs261291, rs727857, rs1047891, rs12794714, rs72997623, rs7528419, rs75741381, rs6782190 and rs77924615 | 0.013 |
| Unclassified | rs6782190, rs61891388, rs12803256, rs1047891, rs212100, rs7522116, rs78151190, rs261291, rs12056768 and rs1933064 | 0.005 |

^1^Likelihood ratio test comparing the besting-fitting fractional polynomial model of degree 1 against the linear model

^2^the nonlinear MR p-value were 0.04, for the GRS excluding the blood traits varaints but keeping the rs1352846 (GC variant).

For TBV/GMV/WMV**:**

**393,425** no TBV data

**742** outside the ±3SD (outliers brain volume data)

For HV

**393,439** no HV data

**740** outside the ±3SD (outliers brain volume data)

For WMH

**394,607** no WMH data

**716** outside the ±3SD (outliers brain volume data)

**Up to 33,523** participants for **observational association** analyses on brain neuroimaging data

**33,523** for TBV/GMV/WMV

**33,511** for HV

**32,367** for WMH

**3,714** of >2 members from a related family excluded

**53,845** participants with no 25(OH)D info

**10,002** participants with 25(OH)D data from aliquot 3

**502,504** full cohort UKB participants**^1^**

**434,943** participants with complete data on 25(OH)D

**427,690** participants for **observational** **association** analysis on disease outcomes

**3,414** incident dementia cases and **424,276** controls

**5,339** incident stroke cases and **422,351** controls

**443** prevalent dementia cases excluded

**6,810** prevalent stroke cases excluded

**133,176** excluded due to

**4,785** No genetic data

**316** mismatch between self-report and genetically determined sex

**67,753 not from** white British **ancestry**

**60,322** related individuals

**294,514** participants for **Mendelian randomization** analysis on disease outcomes

**2,399** incident dementia cases and **292,175** controls

**3,760** incident stroke cases and **290,754** controls

**9,622** for TBV**; 9,620** for HV; and **9,284** for WMH excluded due to

**332 | 332 |** **321** (N are for TBV, HV and WMH respectively) No genetic data

**23 | 23 | 23** sex mismatches between self-report and genetically determined

**4,272 | 4,271 | 4,098** non-white British

**4,995 | 4,994 | 4,842** all related individuals

**Up to 23,901** participants for **Mendelian randomization** analysis on brain neuroimaging data

**23,901** for TBV/GMV/WMV

**23,891** for HV

**23,083** for WMH

**Supplementary Figure 1:** Flow diagram showing the analytical sample size for the observational and mendelian randomisation analysis (diagram in dotted box) involving total and regional brain volumes, dementia, and stroke outcomes. **^1^** actively consented UK Biobank participants. Total brain volume (TBV), Grey matter volume (GMV), White matter volume (WMV), White matter hyperintensity volume (WMH), Hippocampal volume (HV), Standard deviation (SD). Please note the observational analysis were done in all ethnic groups but the mendelian randomization was done on white British participants only.


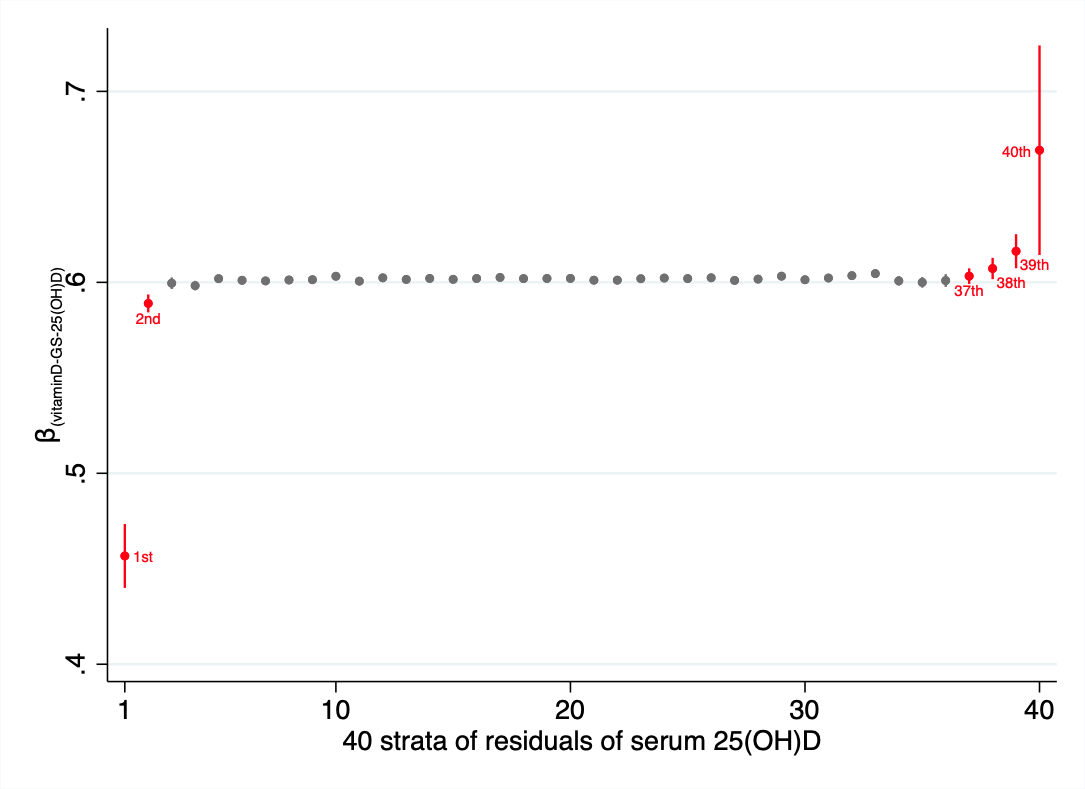


**Supplementary Figure 2:** The estimates from the association between 25(OH)D and genetic score (vitamin-GRS-25(OH)D) across 40 residual 25(OH)D-based strata. Red dots indicate the outliers, and the red lines indicate the confidence intervals of the outliers.


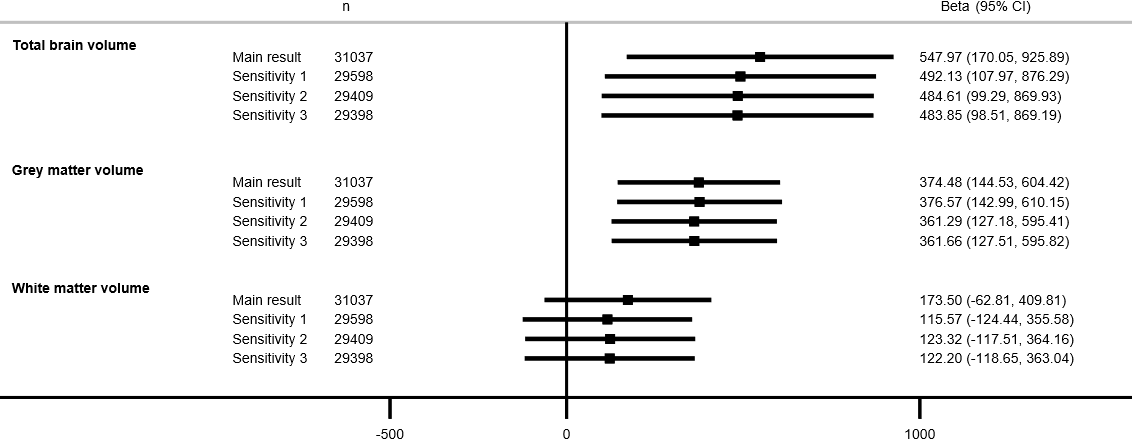


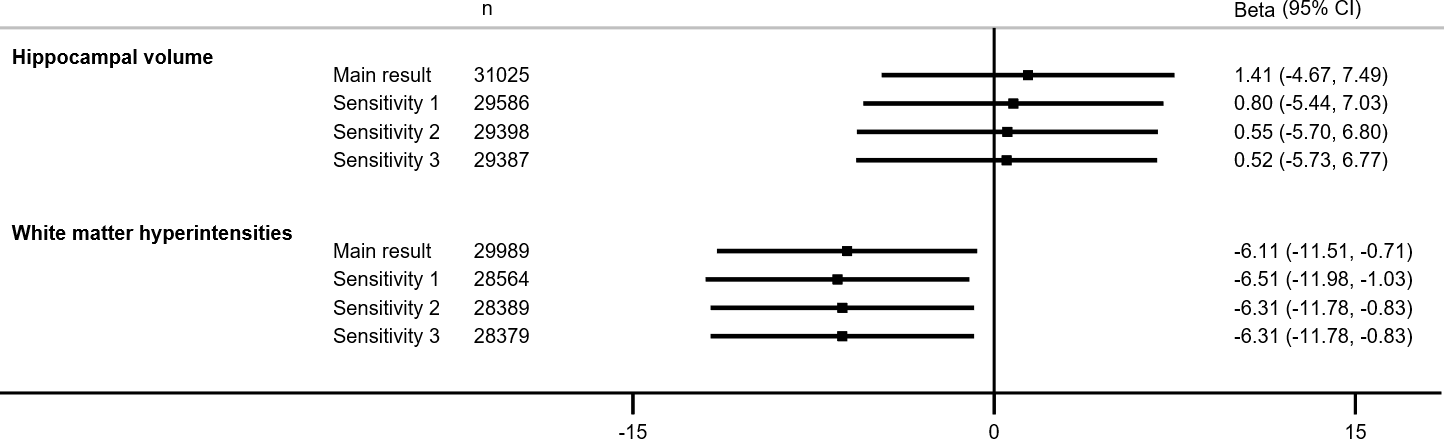


**Supplementary Figure 3**: The observational association of 25(OH)D with brain imaging outcomes for the full model in the main analysis (main result), and three scenarios of sensitivity analysis. Sensitivity 1 after further adjusting for duration between baseline and imaging visits; Sensitivities 2 and 3 are after excluding stoke, and both stroke and dementia that occurred before the imaging visit, respectively.

**
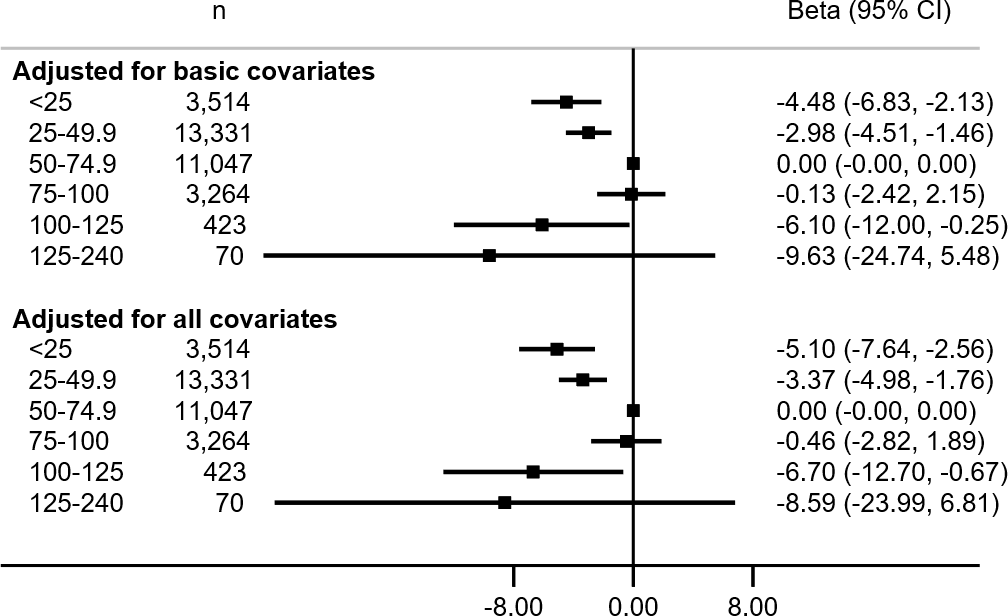
**

**A: Total brain volume**

**B: Grey Matter Volume**

**
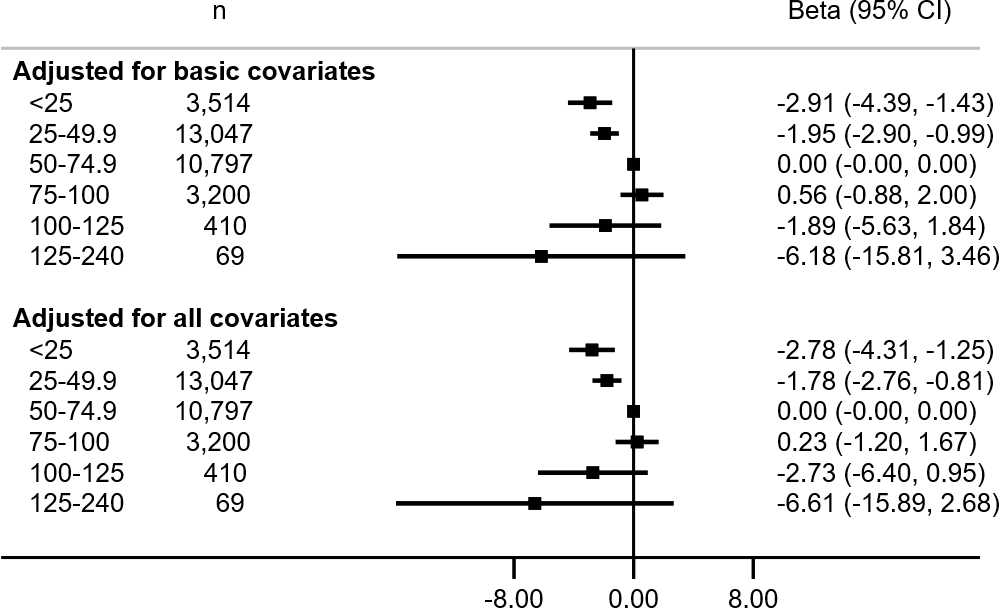
**

**C: White Matter Volume**

**
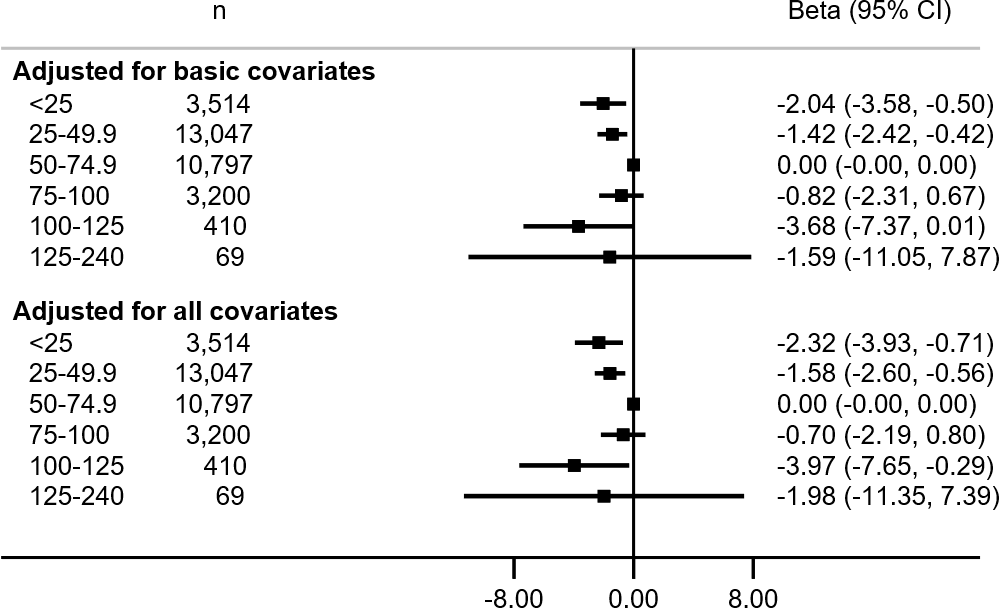
**

**D: Hippocampal Volume**

**
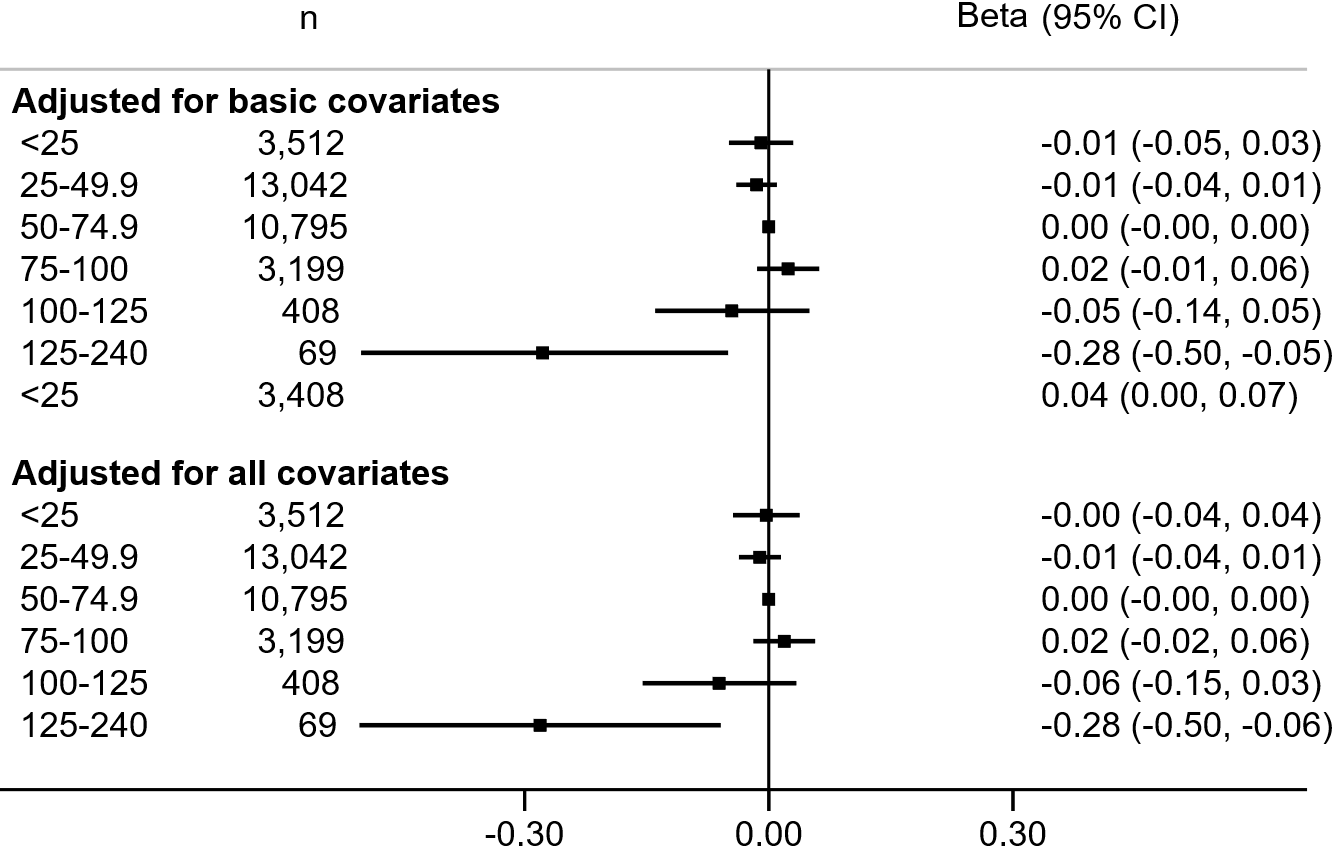
**

**
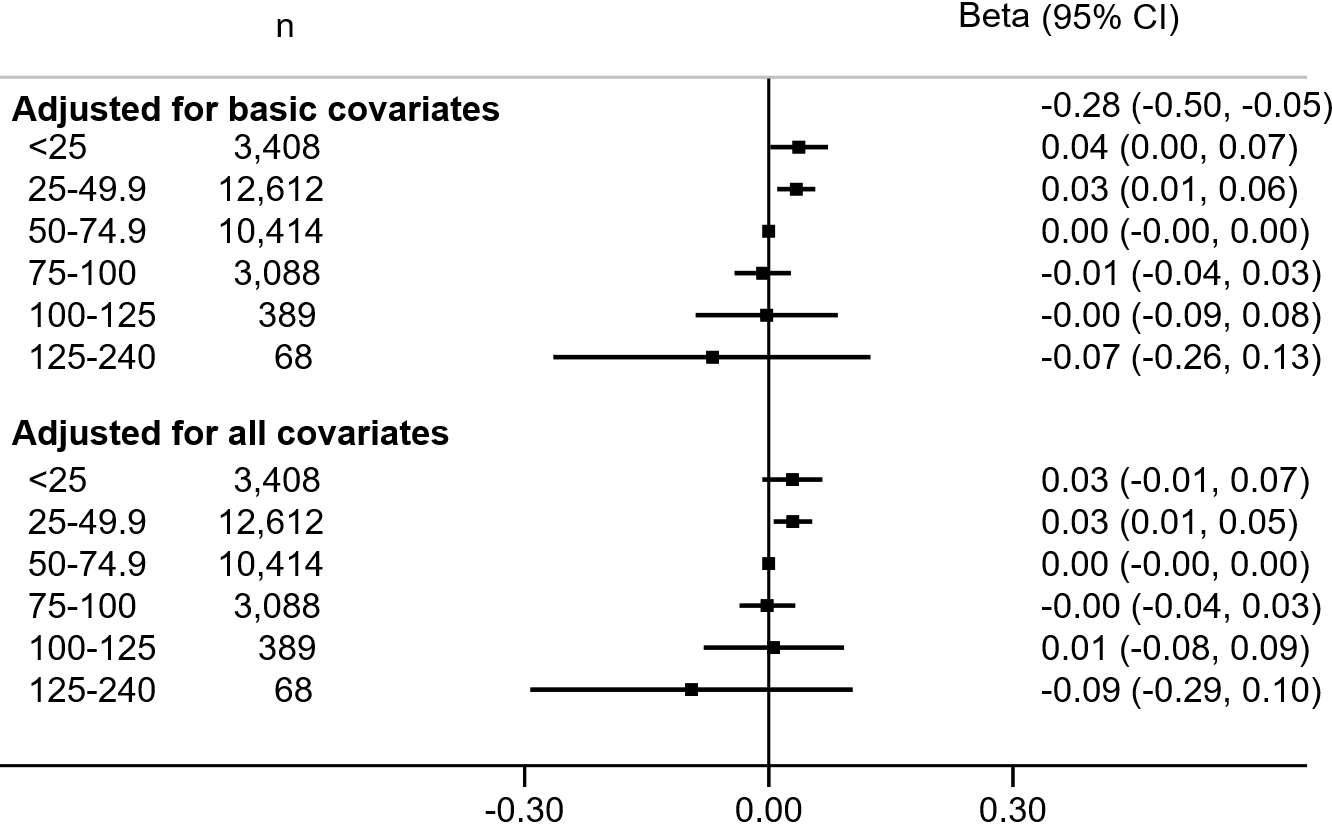
**

**Supplementary Figure 4:** Association between categories of 25(OH)D and Total brain volume (panel A), Grey matter volumes (panel B), White matter volumes (panel C), Hippocampal volumes (panel D) and White matter hyperintensities (panel E), using linear regression, with 50-74.9nmol/L of 25(OH)D as reference. Confidence interval (CI)

**Basic covariates:** age, sex, assessment centre, ethnicity, month
**All covariates:** adjustments made for basic, socioeconomic (education, Townsend deprivation index, employment status), lifestyle (type of physical activity, diet quality, any use of dietary supplements), sun behaviours (time spent outdoors in summer, time spent outdoors in winter, sun protection), and illness (long-standing illnesses and depression) covariates


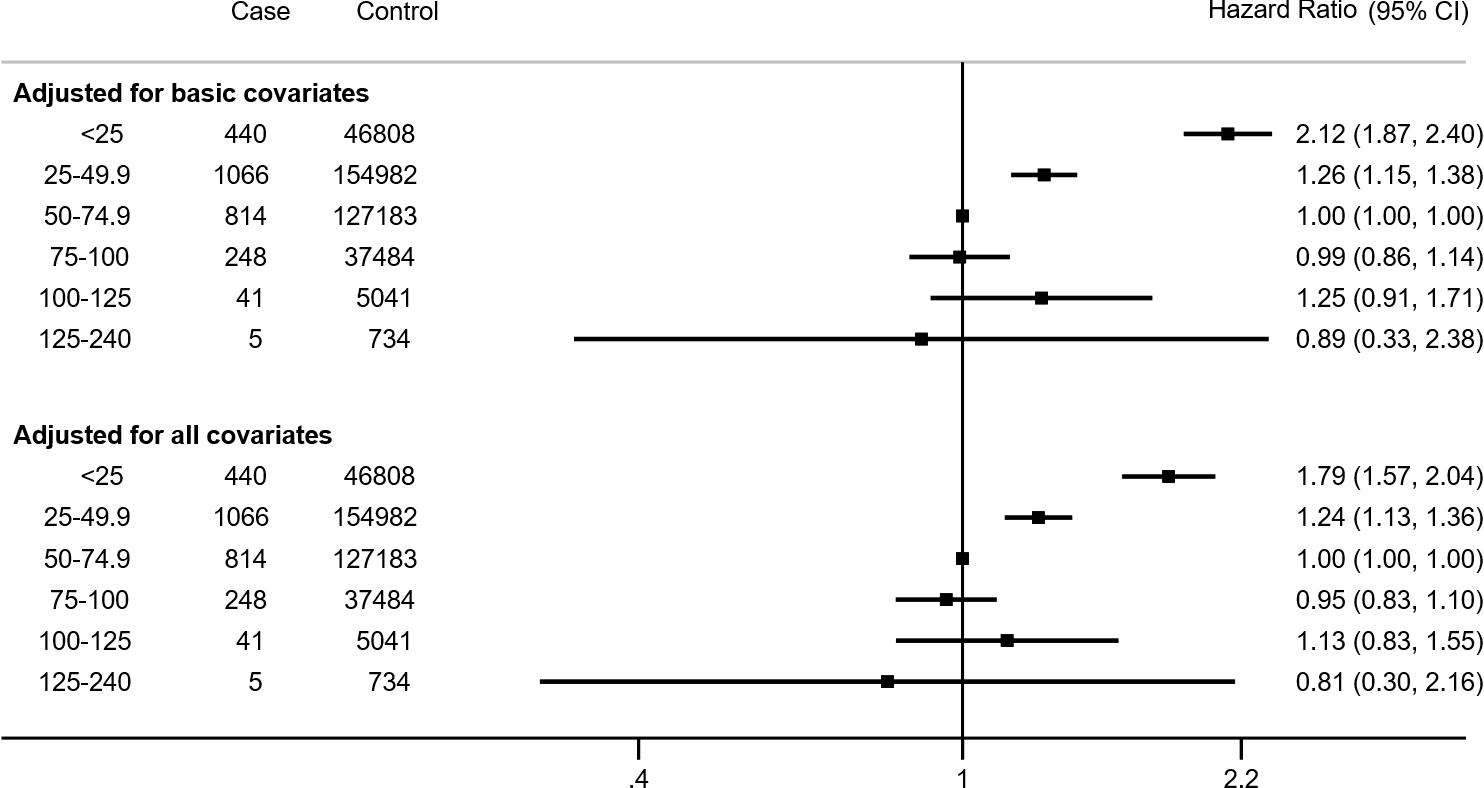


**A: Dementia risk**

**B: Stroke risk**


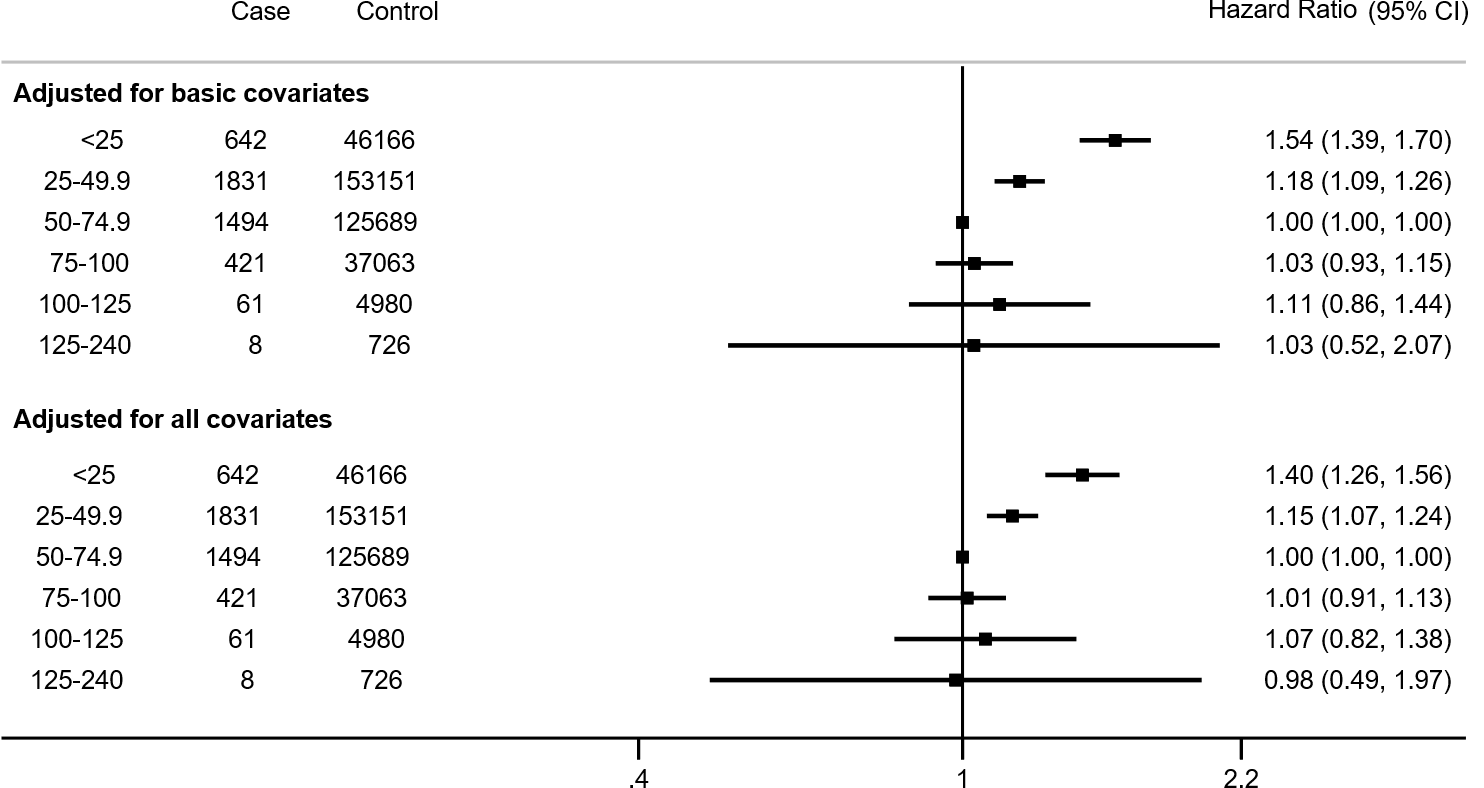


**Supplementary Figure 5:** Association between categories of 25(OH)D and risk of dementia (panel A) and stroke (panel B), using cox proportional hazards model, with 50-74.9nmol/L of 25(OH)D as reference. Confidence interval (CI).

**Basic covariates:** age, sex, assessment centre, ethnicity, month
**All covariates:** adjustments made for basic, socioeconomic (education, Townsend deprivation index, employment status), lifestyle (type of physical activity, diet quality, any use of dietary supplements), sun behaviours (time spent outdoors in summer, time spent outdoors in winter, sun protection), and illness (long-standing illnesses and depression) covariates


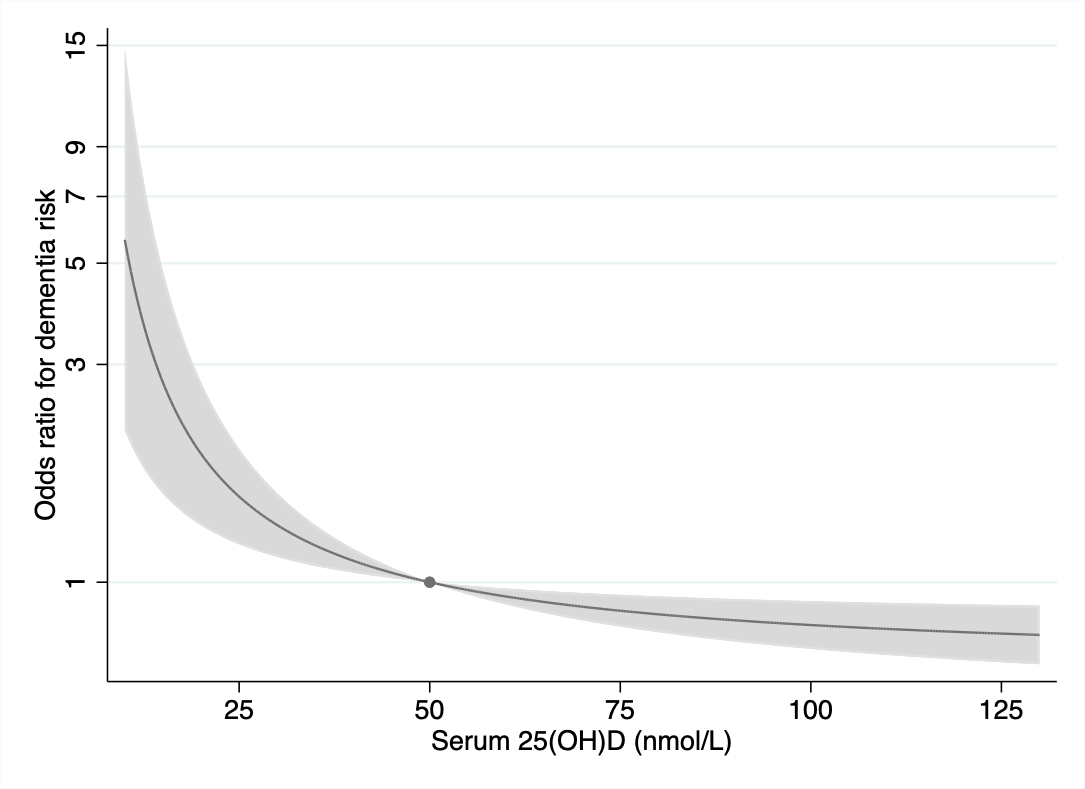


**Supplementary Figure 6:** Association between genetically determined 25(OH)D and odds ratio of dementia using a fractional polynomial model before removing outlying strata (1^st^,2^nd^, 37^th^,38^th^, 39^th^, and 40^th^ Supplementary Figure 2). The dot represents the reference point which is at 50nmol/L and the shaded areas represent the 95% CI.


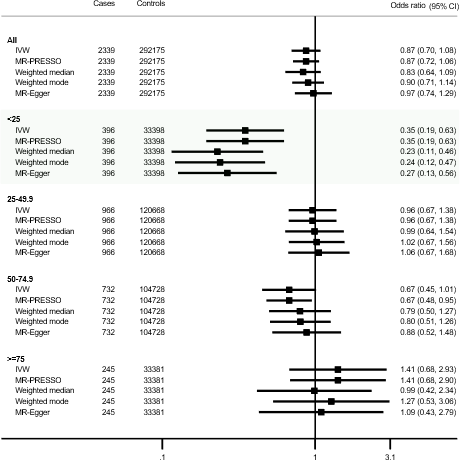


**Supplementary Figure 7:** Overall unstratified and stratified causal linear associations between 25(OH)D and risk of dementia using Inverse Variance Weighted (IVW), mendelian randomization pleiotropy residual sum and outlier (MR-PRESSO), Weighted median, Weighted mode, MR-Egger methods of TwoSample MR analysis. Hazard ratios are per log-transformed 25(OH)D [We used variant-25(OH)D association estimates for the two-sample MR analysis from 25(OH)D Genome Wide Association Study (GWAS) that apply log transformation for 25(OH)D data].

$$\mathrm{PIF}=\frac{\int_{\min}^{\max} P\left( x \right)\mathrm{RR}\left( x \right)\mathrm{dx} - \int_{\min}^{\max} P\left( x^{'} \right)\mathrm{RR}\left( x^{'} \right)\mathrm{dx}^{'}}{\int_{\min}^{\max} P\left( x \right)\mathrm{RR}\left( x \right)\mathrm{dx}}$$


**Supplementary Figure 8:** The potential impact of increasing serum 25(OH)D to different thresholds on dementia risk reduction estimated based on data in the UK Biobank. For instance, 17% (95%CI 7.22, 30.58) of dementia could be prevented in this population by increasing the serum 25(OH)D to 50nml/L for those with serum values below this threshold. Potential impact fractions (PIF), Confidence interval (CI).

References:

1. Manichaikul A, Mychaleckyj JC, Rich SS, Daly K, Sale M, Chen WM. Robust relationship inference in genome-wide association studies. Bioinformatics (Oxford, England) 2010;26(22):2867-73. doi: 10.1093/bioinformatics/btq559.

2. Price AL, Zaitlen NA, Reich D, Patterson N. New approaches to population stratification in genome-wide association studies. Nat Rev Genet 2010;11(7):459-63. doi: 10.1038/nrg2813.

3. Fry D, Almond R, Moffat S, Gordon M, Singh P. Companion document to accompany serum biomarker data. Version current 11 March 2019. Internet: <https://biobank.ndph.ox.ac.uk/showcase/showcase/docs/serum_biochemistry.pdf> (accessed 29 July 2021).

4. UK Biobank. Blood sample collection, processing and transport. Version current 15 April 2011. Internet: <https://biobank.ctsu.ox.ac.uk/crystal/crystal/docs/Bloodsample.pdf> (accessed 29 May 2020).

5. Smith SM, Alfaro-Almagro F, Miller KL. Brain imaging documentation. Version current January 2020. Internet: <https://biobank.ctsu.ox.ac.uk/crystal/crystal/docs/brain_mri.pdf> (accessed 14 April 2020).

6. Alfaro-Almagro F, Jenkinson M, Bangerter NK, Andersson JLR, Griffanti L, Douaud G, Sotiropoulos SN, Jbabdi S, Hernandez-Fernandez M, Vallee E, et al. Image processing and Quality Control for the first 10,000 brain imaging datasets from UK Biobank. NeuroImage 2018;166:400-24. doi: <https://doi.org/10.1016/j.neuroimage.2017.10.034>.

7. Revez JA, Lin T, Qiao Z, Xue A, Holtz Y, Zhu Z, Zeng J, Wang H, Sidorenko J, Kemper KE, et al. Genome-wide association study identifies 143 loci associated with 25 hydroxyvitamin D concentration. Nature communications 2020;11(1):1647. doi: 10.1038/s41467-020-15421-7.

8. Jiang X, O'Reilly PF, Aschard H, Hsu YH, Richards JB, Dupuis J, Ingelsson E, Karasik D, Pilz S, Berry D, et al. Genome-wide association study in 79,366 European-ancestry individuals informs the genetic architecture of 25-hydroxyvitamin D levels. Nature communications 2018;9(1):260. doi: 10.1038/s41467-017-02662-2.

9. Gkatzionis A, Burgess S. Contextualizing selection bias in Mendelian randomization: how bad is it likely to be? Int J Epidemiol 2018. doi: 10.1093/ije/dyy202.

10. Staley JR, Burgess S. Semiparametric methods for estimation of a nonlinear exposure-outcome relationship using instrumental variables with application to Mendelian randomization. Genet Epidemiol 2017;41(4):341-52. doi: 10.1002/gepi.22041.

11. Bowden J, Del Greco MF, Minelli C, Davey Smith G, Sheehan N, Thompson J. A framework for the investigation of pleiotropy in two-sample summary data Mendelian randomization. Stat Med 2017;36(11):1783-802. doi: 10.1002/sim.7221.

12. Bowden J, Davey Smith G, Haycock PC, Burgess S. Consistent estimation in mendelian randomization with some invalid instruments using a weighted median estimator. Genet Epidemiol 2016;40(4):304-14. doi: 10.1002/gepi.21965.

13. Hartwig FP, Davey Smith G, Bowden J. Robust inference in summary data Mendelian randomization via the zero modal pleiotropy assumption. Int J Epidemiol 2017;46(6):1985-98. doi: 10.1093/ije/dyx102.

14. Bowden J, Davey Smith G, Burgess S. Mendelian randomization with invalid instruments: effect estimation and bias detection through Egger regression. Int J Epidemiol 2015;44(2):512-25. doi: 10.1093/ije/dyv080.

15. Verbanck M, Chen CY, Neale B, Do R. Detection of widespread horizontal pleiotropy in causal relationships inferred from Mendelian randomization between complex traits and diseases. Nat Genet 2018;50(5):693-8. doi: 10.1038/s41588-018-0099-7.

16. Kamat MA, Blackshaw JA, Young R, Surendran P, Burgess S, Danesh J, Butterworth AS, Staley JR. PhenoScanner V2: an expanded tool for searching human genotype-phenotype associations. Bioinformatics 2019;35(22):4851-3. doi: 10.1093/bioinformatics/btz469.

17. Zhou A, Selvanayagam JB, Hyppönen E. Non-linear Mendelian randomization analyses support a role for vitamin D deficiency in cardiovascular disease risk. Eur Heart J 2021. doi: 10.1093/eurheartj/ehab809.

18. Novak G, Einstein SG. Chapter 4 - structural magnetic resonance imaging as a biomarker for the diagnosis, progression, and treatment of alzheimer disease. In: McArthur RA, ed. Translational Neuroimaging. 1st ed. London: Academic Press, 2013:87-129.

19. Vemuri P, Jack CR. Role of structural MRI in Alzheimer's disease. Alzheimer's Research & Therapy 2010;2(4):23. doi: 10.1186/alzrt47.

20. Banerjee D, Muralidharan A, Hakim Mohammed AR, Malik BH. Neuroimaging in Dementia: A Brief Review. Cureus 2020;12(6):e8682-e. doi: 10.7759/cureus.8682.

21. Dicks E, Vermunt L, van der Flier WM, Visser PJ, Barkhof F, Scheltens P, Tijms BM. Modeling grey matter atrophy as a function of time, aging or cognitive decline show different anatomical patterns in Alzheimer's disease. NeuroImage: Clinical 2019;22:101786. doi: <https://doi.org/10.1016/j.nicl.2019.101786>.

22. Hase Y, Horsburgh K, Ihara M, Kalaria RN. White matter degeneration in vascular and other ageing-related dementias. J Neurochem 2018;144(5):617-33. doi: 10.1111/jnc.14271.

23. Rutten-Jacobs LC, Larsson SC, Malik R, Rannikmäe K, Sudlow CL, Dichgans M, Markus HS, Traylor M. Genetic risk, incident stroke, and the benefits of adhering to a healthy lifestyle: cohort study of 306 473 UK Biobank participants. BMJ 2018;363:k4168.

24. Pham K, Mulugeta A, Zhou A, O'Brien JT, Llewellyn DJ, Hyppönen E. High coffee consumption, brain volume and risk of dementia and stroke. Nutr Neurosci 2021:1-12. doi: 10.1080/1028415x.2021.1945858.

25. UK Biobank. UK Biobank data showcase. Version current 25 July 2021. Internet: <http://biobank.ctsu.ox.ac.uk/crystal/search.cgi> (accessed 4 June 2020).
